# Mini/Micro/Nano Scale Liquid Metal Motors

**DOI:** 10.3390/mi12030280

**Published:** 2021-03-08

**Authors:** Li Liu, Dawei Wang, Wei Rao

**Affiliations:** 1 Key Laboratory of Cryogenics, Technical Institute of Physics and Chemistry, Chinese Academy of Sciences, Beijing 100190, China; liuli19@mails.ucas.ac.cn (L.L.); wangdawei181@mails.ucas.ac.cn (D.W.); 2 Beijing Key Lab of CryoBiomedical Engineering and Key Lab of Cryogenics, Beijing 100190, China; 3 School of Engineering Science, University of Chinese Academy of Sciences, Beijing 100039, China; 4 School of Future Technology, University of Chinese Academy of Sciences, Beijing 100049, China

**Keywords:** liquid metal, micro/nano scale, motors, transformability, propulsion

## Abstract

Swimming motors navigating in complex fluidic environments have received tremendous attention over the last decade. In particular, liquid metal (LM) as a new emerging material has shown considerable potential in furthering the development of swimming motors, due to their unique features such as fluidity, softness, reconfigurability, stimuli responsiveness, and good biocompatibility. LM motors can not only achieve directional motion but also deformation due to their liquid nature, thus providing new and unique capabilities to the field of swimming motors. This review aims to provide an overview of the recent advances of LM motors and compare the difference in LM macro and micromotors from fabrication, propulsion, and application. Here, LM motors below 1 cm, named mini/micro/nano scale liquid metal motors (MLMTs) will be discussed. This work will present physicochemical characteristics of LMs and summarize the state-of-the-art progress in MLMTs. Finally, future outlooks including both opportunities and challenges of mini/micro/nano scale liquid metal motors are also provided.

## 1. Introduction

Motors and machines play a major role in the development and evolution of modern industry. Since 1959, when Richard Feynman predicted the flourishing research field of nanoscience and nanotechnology, abundant research about the miniaturization of motors and machines to micro/nanoscale have been executed. Micro/nanomotors (MNMTs) have been developed to perform delicate tasks in micro/nanoscale from drug/cargo delivery, tumor therapy, biodetoxification, precision surgery [1] to environmental remediation [2]. These applications are based on the autonomous motion of MNMTs that can convert chemical or physical energy into kinetic energy. In fact, the performance of MNMTs mostly relies on the intrinsic features of adopted materials. At first, most MNMTs are made of metals and metal oxides such as Au, Pt, ZnO, and Cu_2_O to achieve propulsion by a chemical gradient in hydrogen peroxide (H_2_O_2_) [3]. When applied in biomedicine, the chemical fuel is toxic to human. Furthermore, most of them are rigid and inflexible. It is easy to harm the soft tissue in the human body where intricate and soft channels are present everywhere. In addition, polymer-based and bio-hybrid MNMTs have good biocompatibility and low toxicity. However, low stability, fast degradation, and quick clearance are other hurdles for their long-time use. To address these challenges, there is still a strong desire for the incorporation of attractive materials to further expand the application fields of MNMTs.

The room temperature liquid metals (LMs) are a unique group of metals that behave like liquids near room temperature. The pure LMs include cesium (Cs, melting point = 28.5 °C), francium (Fr, 27 °C), rubidium (Rb, 39.3 °C), mercury (Hg, −38.8 °C), and gallium (Ga, 29.8 °C). Among these types of LMs, Hg is highly toxic, Cs and Rb are very reactive, and Fr is radioactive. In comparison, Ga and Ga-based alloys such as EGaIn (75.5 wt% gallium and 24.5 wt% indium) and Galinstan (68.5 wt% gallium, 21.5 wt% indium, and 10 wt% tin), have high chemical stability and negligible toxicity. The Ga-based LMs have recently shown great values in various scopes of applications owing to their unique properties (Table 1), such as basic metallic characteristics (high thermal conductivity, good electrical conductivity, radiopacity, and electromagnetic properties), amorphous properties (superb fluidity, excellent flexibility, shape transformability, self-healing capability, reconfigurability, and low viscosity), and several featured properties (facile functionalization accessibility, good biocompatibility, biodegradability, low toxicity, catalytic properties, photothermal/photodynamic capability, and stimuli responsiveness). Because of these unique properties, LMs hold a great possibility to supplement the vacant applications of conventional materials-based MNMTs, such as microfluidics [4,5], repairing nanonetwork [6], imaging [7], cancer-targeting [8,9], vascular embolism, and so on [10,11].

**Figure 1:**
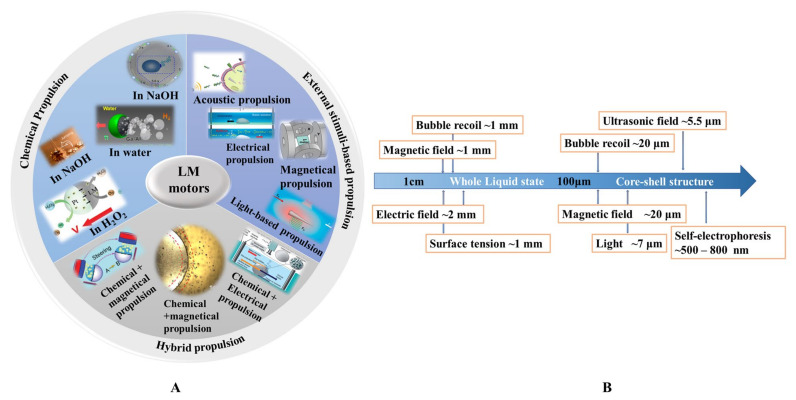
The propulsion mechanism of LM motors. (**A**) LM motors propelled by different energy sources. (**B**) The relationship between the size of LM motors and corresponding driving force.

Liquid metal motors can be powered by various energy sources, including chemical fuels (water, NaOH solution, and H_2_O_2_ solution), external stimuli (electrical, acoustic, magnetic, and light fields), and hybrid sources (Figure 1A). A brief introduction of different propulsion methods of liquid metal motors is summarized in Table 2. Under the motivation of these driving sources, liquid metal motors can not only perform a directional movement, circular motion, and self-rotation, but also a unique large-scale deformation. It is worth mentioning that the mechanisms behind the movement and deformation of LM motors highly depend on the size of LM. Figure 1B shows the relationship between the size of LM and the corresponding driving force. For macroscopic LM motors, the large-scale deformations induced by surface tension are very intriguing [38,39], but the corresponding propulsion is still a challenge due to the requirement of the large driving force. Additionally, the definition of motors emphasizes the significance of directional translational motion [40]. As the size of the LM decreases to less than 1 cm, the propulsion has been successfully and extensively reported. Thus, LM motors below 1 cm, named mini/micro/nano scale liquid metal motors (MLMTs) (MCLMTs for micro/nano LM motors; MILMTs for mini LM motors) will be discussed in this paper. When the size of MLMT is between 1 cm and 1 mm, it is still in a fully liquid state, so it can realize a self-deformation and adapt to its surroundings [41]. At this length scale, the movement of MLMTs requires less driving force, and the main driving force is bubbles recoil force and surface tension. Once the size of the LM declines to the micro-nano field, asymmetric structure designs of LM are essential to achieve effective propulsion. Although the surface oxide makes the MLMTs in a core-shell structure, it is helpful to maintain its shape, separate state, and prevent fusion [6,8,25], as it can also cause deformation. So far, although great progress has been achieved in MLMTs, it is still a significant challenge to quickly mass-produce MLMTs, precisely control the motion, perform more delicate tasks, and achieve more potential applications. Based on the extensive exploration of the tremendous ubiquitous characteristics of LM over past decades, there are vast opportunities to endow motors with new vitality and impart fresh life in more possible filed by combining the prominent properties of LM with MNMTs.

In this paper, we will first introduce the properties of micro/nanoscale LMs to guide researchers interested in the utilization of LMs to meet their specific needs. Then, the preparation, driving mechanism, and application of MLMTs are summarized. In particular, the different principles and methods behind the fabrication and propulsion of MLMTs of different sizes will be highlighted in this part. Lastly, the challenge and future outlook will be given.

## 2. Physicochemical Characteristics of Micro/Nanoscale LMs

LM micro/nanomaterials (LMMMs), especially LM micro/nanoparticles (LMNPs), pose unique physicochemical characteristics and wide applications, which will endow novel properties and functions to motors. Here, the properties of LMMMs related to motors will be discussed for better utilization of LM in MNMTs. More specific investigation and discussion about LMNPs such as surface microenvironment modification [42], nanocomposite [27], and biomedical [43] or chemical [44] applications can be found in recent reviews.

### 2.1. Shape

**Figure 2:**
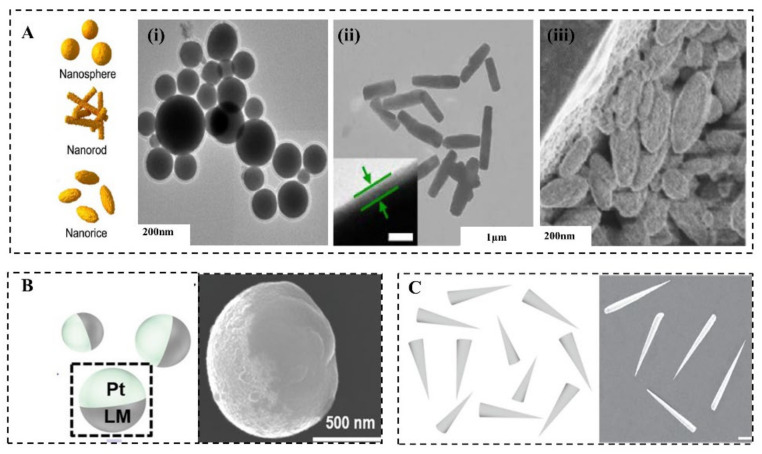
Shapes of LM micro/nanomaterials (LMMMs) and motors. (**A**) Different shapes of LM nanomaterials. (**i**) Transmission electron microscopy (TEM) of eutectic gallium indium liquid alloy micro-and nanoparticles [45]. (**ii**) TEM of EGaIn alloy rods [25]. (**iii**) SEM image of EGaIn nanorices [47]. The asymmetric shape of MCLMTs: (**B**) Janus sphere [6] and (**C**) needle [8].

LMMMs are generally in the shape of spheres [45], rods [46], and rice (Figure 2A) [47], which is determined by fabrication methods and parameters [46,48]. There are three common methods to prepare MCLMTs: Sonication [25], transfer printing [49], and pressure-filter-template [9] methods. The former one can produce LMMMs in the sphere, rod, and rice shape by varying parameters. The latter two can prepare the sphere, rod, or needle, and even dumbbell-shaped LMMMs by changing templates. For the sonication method, different parameters lead to various shapes of the LM nanostructure. The ultrasonication time [50], temperature [51], medium [46], and additive [45,47,51,52] affect the shape and size of LMMMs significantly. The rod shape formation can be attributed to the formation of flake oxide GaO(OH) [50], in the presence of dissolved oxygen with a consistently high temperature induced by ultrasonication. Further, the equation for GaO(OH) formation can be given by [50]:

(1) $$2{\mathrm{Ga}}_{\left(1\right)}+2{\mathrm{OH}}_{\left(\mathrm{aq}\right)}^{}+O_{2(\mathrm{aq})}\to 2\mathrm{GaO}{\left(\mathrm{OH}\right)}_{\left(s\right)}$$

In addition to the ultrasonic method, LM nanorods can also be prepared by the pressure-filter-template method [8,9]. It should be noted that this method produces nanorods by generating surface oxides of Ga_2_O_3_ instead of GaO(OH) mentioned above because the reaction of Ga with oxygen at mild temperatures forms Ga_2_O_3_. Additionally, Ga_2_O_3_ on the surface is necessary to stabilize the rod shape because of the inner amorphous liquid Ga.

The shape of LMMMs highly affects the effective propulsion and function of MCLMTs [40], thus should be carefully considered to meet specific requirements in various potential applications. It is worth mentioning that the relation between the shape or structure and propulsion mechanism is universal to MNMTs of other materials, and more information can be found in these reviews [3,53,54]. Fundamentally, the shape of LMMMs significantly influences the propulsion mechanism [40]. For instance, the Janus-sphere constructed by applying other materials such as Pt on half of it (Figure 2B) can be propelled in H_2_O_2_ by bubbles ejected from one side of it [6]; rods with two ends that differ in size are the prerequisite for MCLMTs to achieve movement by producing enough pressure difference along the long axis under the ultrasonic field or light field (Figure 2C). When increasing the size difference of two ends, this rod motor can achieve a higher speed than the normal rod one [9]. By contrast, spheres can be hardly propelled by these two fields, because the symmetry lengths of diameters cannot produce enough pressure difference. For MCLMTs motivated by magnetic fields, special designs such as helical tails and dumbbell-like shapes are required [49]. Besides, the function of LMNPs can also be influenced by their shape. The LM nanomotors in rod [9] and needle [8] shape are more likely to be developed to open the cell membrane mechanically. Compared with the spherical counterpart, they can move in a rotating way and have a smaller contact area with the cell. Furthermore, these rod and needle LM nanomotors would transform into spheres in the acid environment such as endosomes [9], which is beneficial for further fuses and degrades in cells. Moreover, LMNPs can disrupt the endosomal membrane to achieve effective drug delivery by the transformation to the hollow rod by light irradiation [20]. Another similar transformation is utilized to break the bacterial biofilm, which can achieve a 99% killing rate of bacterial [22].

### 2.2. Electrical/Thermal Properties

LMNPs are normally non-conductive because of their semi-conductive Ga_2_O_3_ oxide skin. However, external stimuli could remove or break this oxide and high conductivity can be regained. Many methods are adapted to form a highly conductive path, including laser sintering [55], mechanical sintering [46], and peeling off [56]. During these methods, mechanical sintering [57] is a facile way to induce a conductive path by introducing particle interface rupture, allowing the inner LM to flow out to coalesce into a continuous liquid conductive circuit. It is operated at room temperature without requiring any complex equipment. In addition, the acid vapor [58] and mechanical fracturing [59] can be used to remove the LM oxide and achieve the conductive pathway as well. For example, Kim et al. proved that the hydrochloric acid (HCl) vapor can be applied to react with the oxide skin of Galinstan (Ga_2_O_3_ and Ga_2_O) and change it into InCl_3_ and GaCl_3_, which poses a better conductivity than the initial LM particles [58]; Wang et al. used this method to remove the oxide skin on LM Janus nanomotors to achieve a conducive state, thus having the function of repairing silver nanowire networks [6].

The melting point of various LMNPs is generally influenced by their alloying elements and size. Ga and most LM have low melting points and high boiling points, keeping them in the liquid state across a wide temperature range (8–2200 °C). Generally, the melting point of Ga-based alloy is even lower than pure Ga and is influenced by the composition ratio of different elements [44]. Moreover, the size of LMNPs is proven to change the melting point of LM significantly [60], as the melting temperature was found to be decreased relative to values of bulk LM. According to Ghigna et al. [55], the analysis of extended X-ray absorption fine structure spectra of Ga particles shows the melting point of LMNPs (diameter ~150 nm) was 300 K, similar to that of the bulk value (303 K), while an approximate 110 K and 20 K decrease was observed for LMNPs with diameters around 10 nm and 30 nm, respectively. Similarly, recent research [48,56] on the size dependence of the melting point of LMNPs supports the aforementioned trends. Based on the supercooling nature and high conductivity of LMNPs, Simge et al. reported a facile heat-free joining technique [59]. This technology breaks the outer oxide layers of LMNPs through mechanical stressing or shearing, thus triggering the inside fluid flow accompanied by deformation, alloying, and solidification.

### 2.3. Optical Properties

Localized surface plasmon resonance (LSPR) is a surface charge density oscillation at certain optical frequencies, which can occur on most kinds of metals. For Ga-based LMNPs, the oscillation frequency of an electron cloud lies in the range of the ultraviolet to the visible light [61,62,63]. Strategies involving particle size and shape control [64,65], surface oxide thickness adjustment [66], alloy composition changing [67], and substoichiometric doping levels of metal oxide on the LM surface [68] can be utilized to control UV-plasmonic of Ga-based LMPs. 

**Figure 3:**
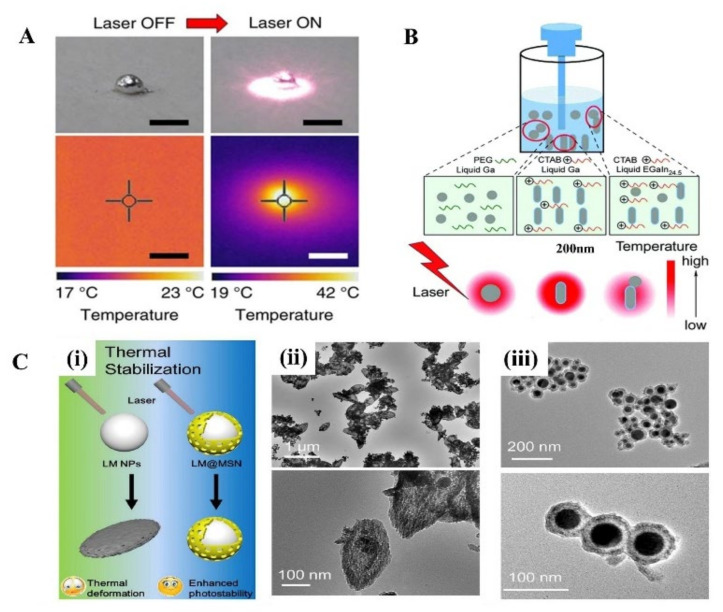
The photothermal effect of LMMMs. (**A**) Visible light and thermographic images of a LM droplet (1 mg) before and after irradiation with a 785 nm NIR laser at 1 W for 5 min. Scale bars, 2 mm [69]. (**B**) Schematic illustration of the photothermal effects of three different kinds of LM nanomaterials [31]. (**C**) (**i**) The scheme of morphology changes of LM and LM@MSN after 808 nm laser irradiation. Representative TEM images of LMNPS (**ii**) and LMNPs@MSN (**iii**) after 808 nm laser irradiation (1 W/cm^2^) for 10 min [34].

In the near-infrared (NIR) light range, although a strong absorption peak has not been observed in LM, it turns out LMNPs have a high photothermal conversion efficacy with the irradiation of NIR light. The photothermal properties of LM treated with NIR light have received increasing attention since the Miyako group’s investigation on it, where they prepared EGaIn nanocapsule encapsulated by DSPE-PEG2000-Amine and DC (8,9) PC with core-shell structure [69]. Under 1 W (80 mW/mm^2^) irradiation with a 785 nm NIR laser, it shows a quick temperature rise, superior photothermal conversion efficiency, and photothermal stability. The surface temperature of the 1 mg LM droplet quickly rose from 20 °C to 43 °C after 5 min irradiation (Figure 3A). In addition, the photothermal conversion efficiency of the LM nanocapsules is almost 3 times that of commercial Au-NR_1_ (52% and 17%, respectively). Moreover, the optical absorbance of the LM capsule can last for 1 h without observed degradation.

Then, a great number of researchers explore the photothermal application in cancer therapy and prove it has an excellent therapeutic effect. Sun et al. systematically evaluated the photothermal properties of various LMMMs, such as Ga nanorods (GaNR), Ga nanospheres (GaNS), and gallium-indium alloy nanorods (LMNR) [31]. Additionally, results show that GaNR exhibited an outstanding photothermal conversion efficiency and distinct temperature elevation compared to GaNS and LMNR. However, the poor thermal stability of pure LMMMs after irradiation impedes their broader applications. Hu et al. proposed a surface mesoporous silica coating strategy on LMNPs (LMNPs@MSN) (Figure 3B) [34], which significantly improved the stability and sustainability of the photothermal performance of LMNPs (Figure 3C). With the modification of HA and loading of doxorubicin (Dox), LMNPs showed significant inhibition of solid tumor growth by the combination of photothermal and chemotherapy both in vitro and in vivo experiments. Similarly, another group proved [35] that LMNPs coated with silica and modified with RGD peptides are an efficient photothermal conversion nano agent. Besides, when LMNPs embedded in poly (NIPAm-co-MBA) hydrogels (PNM) the photothermal conversion efficiency was improved, causing the temperature rise to exceed the shrinkage threshold of the hydrogels, thus promoting the controlled release of drugs (Dox) [32]. In addition, LMNPs and drugs can be loaded on other nanoparticles and show excellent photothermal and chemical synergistic therapy as well [33].

### 2.4. Oxidability

LMNPs easily form a thin oxide skin on the surface even in an extremely low-oxygen environment. The thickness of this oxide skin varies from 0.7 nm to 3 nm [45,52] depending on but not limited to oxygen concentration [70], vapor concentration, surfactant [45,47,70], and oxide-forming time [52]. In most cases, the composition of this oxide skin is mostly Ga_2_O_3_, with a little fraction of In or Sn. The co-existence of LM and this oxide exhibits unique physiochemical properties such as facilitating electron extraction [71], improving photocatalytic activity [72], and providing a strategy for adjusting surface plasmon resonances [66,73]. Besides, the size, shape, morphology, thermal, electrical, and optical properties of LMNPs are influenced by the LM oxide significantly, which is described in the respective sections. Furthermore, LM oxide provides a surface conjugation site for functionalization. Surfactants including thiols [70,74], PEGylating [75], and positive-charged molecules [51] and other strategies such as membrane coating [8] and metal particles coating [76] are successfully assembled on the surface of LM, which is vitally important for the application of LMNPs in biomedicine, on-demand design of MLMTs and other fields. 

Among many surfactants, thiol can not only help to stabilize LMNPs, but also can serve for drug delivery and tumor targeting. Hohman et al. investigated the function of thiol-containing molecules in the process of EGaIn nanodroplets formation [45]. The results show that the thiols further decrease the size of LMNPs and prevent rapid agglomeration, which is attributed to the formation of a metal thiolate complex. Besides, EGaIn functionalized with thiolated (2-hydroxypropyl)-b-cyclodextrin and thiolated hyaluronic acid have the function of targeting drug delivery and enhanced chemotherapeutic inhibition towards the tumor [77]. These two thiols not only cap EGaIn, but more importantly serves as a drug (Dox)-loading and active targeting moiety, respectively. However, there are still some defects for these thiol-terminated surfactants. For example, some thiol groups tend to couple with reactive oxygen species (ROS) and inactivate metalloenzymes in the human body. Many thiols are water-insoluble, which is hard to stabilize LMNPs in water. Apart from thiols, PEGylating provides effective strategies to improve the water-stability of LMNPs. For example, Chechetka prepared LM nanodroplets with PEGylating modification, showing high water dispersibility and no precipitations for at least three days at 20 °C [69]. Sun et al. added thiol-polyethylene glycol-thiol (HS-PEG-HS) into LMNPs preparation solution to obtain well water-dispersed LMNPs, stable for months [31]. Moreover, some surfactants can contribute to the formation of LMNPs in various shapes. For example, melanin helps the formation of rice-shaped LMNPs, which turns out to have the best photothermal conversion efficiency among the LMNPs in the sphere, rod, and rice shape [47]. In addition, lysozyme (positively charged surfactants) play a significant role in the sphere-rod transformation of LMNPs while no rod structure was observed with negatively charged surfactants. 

Other surface modification strategies have also been implemented on LMNPs. The cell membrane can be assembled onto the surface of LMNPs through an acoustically assisted nanovesicle fusion method [8], which enables MLMTs capable of avoiding bio-fouling and actively seeking cancer cells. In addition, the other metal particles can also be added to the surface of the metal to form “liquid marbles”. Galinstan coated with insulators (including Tefl on and silica) and semiconductors (including WO_3_, TiO_2_, MoO_3_, In_2_O_3,_ and carbon nanotubes) have mechanical stability and new functionality [76]. Further, Zhang et al. reported a LM marble with WO_3_ coating in microscale, which has high sensitivity towards low concentrations of heavy metal ions, and enhanced solar light-driven photocatalytic activities [73].

### 2.5. Stimulus–Responsive Transformable Properties 

#### 2.5.1. Acid-Induced Responsive Properties

**Figure 4:**
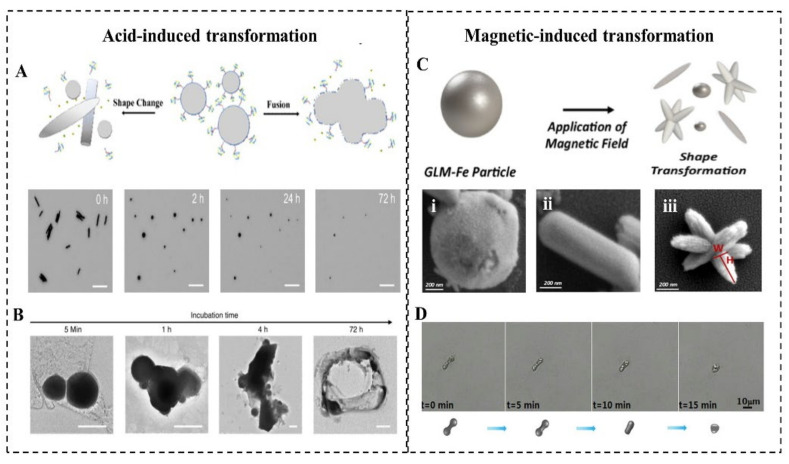
Acid-induced and magnetic-induced morphological transformation. (**A**) Schematic changes and time-lapse images (Scale bars, 10 μm) induced by acid including shape change and fusion of the LMNPs [9,78]. (**B**) Representative TEM images of LM LMNPs loaded with Dox after different incubation times immersed in acidic (pH = 5.0) PBS buffer. The scale bars are 100 nm (for 5 min, 1 h, 4 h) and 400 nm (for 72 h) [77]. (**C**) Schematic changes and SEM images of magnetic Galinstan particle transformed in three morphological categories following magnetization: (**i**) Spheroids, (**ii**) rods, and (**iii**) stars [22]. (**D**) Time-lapse images represents efficient morphological transformation of for micro/nano LM motors (MCLMTs) with an alternating magnetic field (AMF)-irradiation (~20 kHz, average power Pav = 2 kW) [49].

LMMMs with a thin oxide can react with acid slowly, which triggers its transformation (Figure 4A). Lu et al. first investigated the acid-triggered conformational transformation of LMNPs and proposed the potential function of promoting drug release and X-ray imaging [77]. When exposed to an acid environment (pH = 5.0), LMNPs gradually fused within the first 5 min and then aggregated into larger droplets over time. This transformation is induced by the acid removal of oxide Ga_2_O_3_. The inside pristine LMs exits as droplets and quickly merge into large droplets due to the high surface tension when contacting with other nearby droplets. Then the LM microdroplets (LMMDs) were observed to be degraded into the hollow polymeric shells (Figure 4B). The transformability of LMNPs in the acid environment such as tumor sites can significantly promote drug release, which is experimentally proved in Wang’s research [8]. The drug release rate from MCLMTs in the acidic buffer (47.4%) is 8 times higher than that in the neutral buffer (5.3%) within 4 h. Furthermore, the drug release from the membrane-coated MCLMTs can achieve 72.6% in the acidic buffer within 4 h. In comparison, the drug release of rigid metal-based MNMTs mostly rely on external stimuli such as magnetic interaction and ultrasound triggering, and the in-situ acid-triggered drug release of LMNPs does not require complex external equipment and delicate operations. Besides, the fusion of LMNPs accumulated at the tumor site could enhance the contrast of tumor tissue during X-ray imaging. Such acid-induced responsive transformability offers great promise for the future in vivo drug release and cargo delivery fields.

#### 2.5.2. Magnetic-Induced Responsive Properties

The transformation of LMNPs induced by magnetic usually involved the addition of magnetic materials into LM, such as Fe, Ni, and so on. Elbourne et al. reported a magnetic-responsive Galinstan-Fe micro/nanoparticle (GLM-Fe) that can transform from particles to nanorod and nanostars with shaping edges (Figure 4C) [22]. When placed in contact with a bacterial biofilm, GLM-Fe with sharping edges activated by the magnetic field can simultaneously rupture the biofilm matrix and lead to 99% nonviable bacteria. This transformability can be contributed to the liquid nature of the inner core of particles. The drag force experienced by a GLM-Fe when subjected to the magnetic field actuates the physical distortion of particles, leading to an increase of the surface area of LM particles. With local heating induced by the magnetic field, it facilitates the oxidation of the surface to be a nanosheets-shaped GaO(OH) and then rolls up to form rods. Subsequently, the rods aggregate to form the star-like shape with sharping edges. Significantly, the strength of the magnetic field is crucial for this transformation. When GLM-Fe was exposed to a rotating ferrite rare-earth magnet, with a magnetic field strength of 775 milligauss, it shows an obvious morphological change. In contrast, a smaller magnetic field created by a neodymium magnet fails to induce this shape transformation. When the heat is insufficient to promote the oxidation and the drag force is too weak to change the surface area, another type of shape transforms of LM nanomaterial will be triggered [49]. With an alternating magnetic field (AMF) irradiation, the EGaIn MNMTs underwent a morphological transformation from a bowling-pin-like shape to a sphere in an aqueous environment (Figure 4D). It is believed that this transformation is attributed to the local heat generated by eddy currents, resulting in rapturing and removed oxide skin on the LM surface. Then the inner pristine LM flows out to form a droplet due to the high surface tension. 

#### 2.5.3. Temperature-Induced Responsive Properties 

LM with a low melting temperature allows temperature-induced responsive manipulation by temperature variation. The temperature-triggered transformation is more easily achieved in LM than other rigid metals with a much higher melting point. Until now, high and low temperatures have been utilized to induce the transformation of LMNPs and endow their potential in the application of nanomedicine.

**Figure 5:**
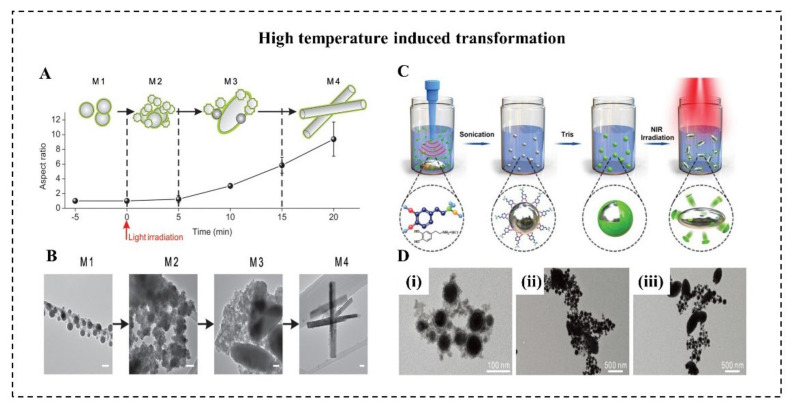
High-temperature-induced morphological transformation. (**A**) Schematic illustration and (**B**) representative TEM images (Scale bars: 100 nm) in the aspect ratio of the LMNPs coated with graphene quantum dots (GQDs) over light irradiation time [20]. (**C**) Schematic illustration of high-temperature-induced morphological transformation polydopamine (PDA)-coated LM microdroplets (LMMDs) [21]. (**D**) TEM images of PDA-coated LMMDs irradiated with NIR laser for (**i**) 0 min, (**ii**) 10 min, and (**iii**) 20 min [21].

The rising temperature on LMNPs can induce the morphology transition from a sphere to a rod. This is attributed to the generation of rod-shaped GaO(OH). Several researchers suggest that heating is a necessary condition for (GaO)OH formation [20,21,51]. When LMMDs in an aqueous solution are heated up to about 70 °C for 30 min, the spherical nanoparticles will be oxidized, leading to the formation of rod-like GaOOH. Moreover, the rising temperature induced by light irradiation can also effectively promote this sphere-to-rod shape transformation. Lu et al. proposed an EGaIn-based LMNPs can undergo a dramatic morphological transformation in an aqueous environment with light irradiation (Figure 5A) [20]. With 635 nm light at a power density of 100 mW/cm^2^, these LMNPs coated with graphene quantum dots (GQDs) gradually experienced the shape transformation from nanoparticles (350 nm) to rod-like nanostructures (900 nm) (Figure 5B). It worth noting that the heat and ROS generated by GQDs with light irradiation created the necessary conditions for oxidizing spherical EGaIn nanoparticles to rod-like GaO(OH). In contrast, LMNPs with light at the same intensity, but without GQD coating, subsequently showed no sign of morphological transition. This sphere-rod transformation turned out to be an effective method to physically disrupt the endosomal membrane thus promoting endosomal escape of payloads. Similarly, Gan et al. reported that LMMDs coated with polydopamine (PDA) (Figure 5C) can transform from sphere to ellipsoid by NIR laser irradiation (Figure 5D) [21]. This transformation depends on the significant heating caused by PDA, which has a high photothermal conversion capability. With 808 nm laser at a power density of 2 W/cm^2^ for 20 min, the temperature of suspension of LMMDs coated with polydopamine (PDA) gradually increased 10 °C, at least 5 °C higher than that of pure LMMDs. Besides, the thickness of the PDA shell should be less than 15 nm to guarantee the shape transformation, because the thicker and stronger PDA shell may prevent oxidation of Ga by impeding oxygen and water diffusion.

**Figure 6:**
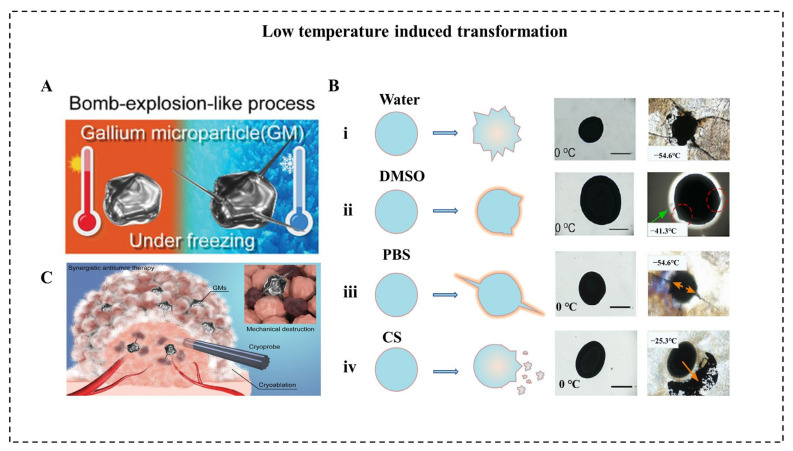
Low-temperature-induced morphological transformation. (**A**) Schematic illustration of low temperature-induced LM bomb-explosion-like transformation [18]. (**B**) Schematic illustration of synergistic antitumor therapy based on cryoablation as well as LM-induced mechanical disruption of tumors [18]. (**C**) Schematic illustration and representative photographs of LM in (**i**) water, (**ii**) DMSO solution, (**iii**) PBS solution, and (**iv**) chitosan (CS) solution [18,79].

The low temperature can also induce LM transformation to achieve various applications. When the ambient temperature is reduced to a low level, the volume of conventional metals tends to decrease due to their positive thermal expansion coefficient. Unlike conventional metals, LM expands in volume after solidification, and the volume expansion ratio of bulk LM was reported to reach 3% [15]. In micro/nanoscale, Sun et al. reported a large-scale transformation of LMMDs with a deformable area increase of 13.8% due to the liquid-solid phase transition in an aqueous solution [79]. As temperature decreased at a speed of 10 °C/s to a final temperature of −60 °C, LMMDs transform from an oval shape into an amorphous shape with sharp edges within 1 ms. In addition, the same group reported a drastic “bomb-explosion-like” deformation of LMMDs in chitosan (CS) or PBS solution (Figure 6A) [18]. Many factors influence the success of shape transformation of LMNPs induced by decreasing temperature, including the types of solution, the size of particles, and the cooling rate. Significantly, the deformation degree of LMNPs varies in various mediums, such as water, dimethyl sulfoxide (DMSO), PBS, and CS solution (Figure 6B). Different solutions influence the formation of ice crystals and the properties of LMMDs, and it is essential for the shape transformation that the LMMDs phase change happened after the ice crystal formation of a solution, leaving little space for deformation. For example, DMSO can inhibit the formation of ice crystals and smooth the edge of the crystals [80] while chitosan solution can enhance the oxidization property and the plasticity of LMMDs. Thus, the deformation behaviors are different in these two solutions. In addition, to guarantee the shape deformation of LMMDs, the cooling rate should be higher than 2 °C/min. This large-scale deformation of LMMDs can be applied to temperature-controlled sensors, which pave the way for human basic equipment to explore extremely cold environments on earth or outer space. Furthermore, this shape transformation can exert mechanical destruction to tumor cells, which shows an enhanced tumor cryoablation effect (Figure 6C).

#### 2.5.4. Toxicity

Although the toxicology profiles of LMNPs have not been thoroughly tested, an increasing number of studies have shown that LMNPs could be biocompatible both in vitro and in vivo.

**Figure 7:**
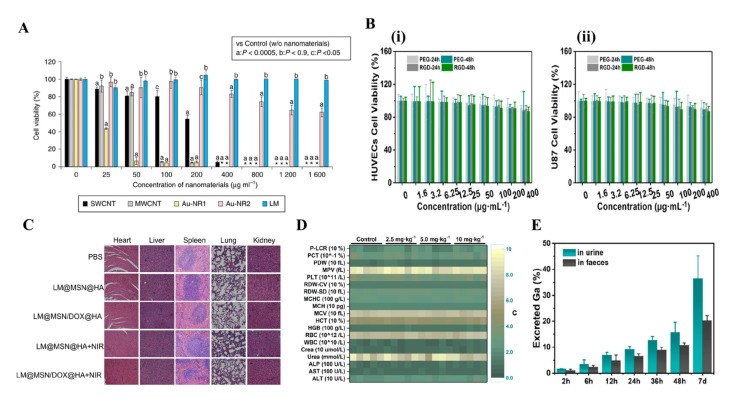
Biological toxicity of LMNPs. In vitro (**A**) Cell viability treated with different nanomaterials, such as single-walled carbon nanotubes (SWCNTs), multi-walled CNTs (MWCNTs), Au-NR_1_ (ethyl-terminated-hydrophilic polymer conjugated gold nanorod), and Au-NR_2_ (methoxy-PEG3000-SH-modified gold nanorod), LMNPs. * means: not determined because all of cells were dead [69]. (**B**) Cell viability of (**i**) normal and (**ii**) cancer cells after treatments with LMNPs coated with SiO_2_ [35]. (**C**) H&E staining images of major organs with different treatment [34]. (**D**) Hematological indexes of the mice sacrificed 30 days post-injection of LMNPs coated with SiO_2_ [35]. (**E**) Cumulative excretion of Ga in feces and urine [35].

In an in vitro experiment, Wang et al. demonstrated that the Ga-LMNPs without any modification have negligible cytotoxicity to both normal cells and cancer cells (over 80% of human L-02 hepatocytes and HeLa cells remained alive at 300 mg/L) [9]. In addition, the cytotoxicity of LMNPs with modification is also investigated by many researchers. Ga-LMNPs encapsulated in glucan particles were demonstrated to have low toxicity to cells, by adding GP-Ga to the murine B6 macrophage cells at a ratio of 10: 1 GP: cell [81]. What is more, compared with other mature but rigid nanomaterials such as single-walled carbon nanotubes (SWCNTs), multi-walled CNTs (MWCNTs), and gold nanorods (Au-NRs), LMNPs displayed lower toxicity at high concentrations (>90% cells were alive at 1600 μg/mL) (Figure 7A) [69]. Recently, other research involving LMNPs also proved its low cytotoxicity at normal treatment dose, such as LM-loaded polymeric hydrogels [32], injectable LM, methotrexate-loaded microsphere [33], and LM coated with SiO_2_ (Figure 7B) [34,35].

In an in vivo experiment, Chechetka et al. demonstrated that high concentrated LMNPs (up to 320 mg/mL) in injections had no negative effect on the viability and body weight of mice up to 19 days [69]. Furthermore, Lu et al. systematically evaluated the toxicity of LMNPs in mice and concluded that LMNPs were highly encouraging for the application of nanomedicine, based on the results that it displayed no obvious toxicity at the treatment dose [77]. Recently, more work on the in vivo toxicity showed the low toxicity of LMNPs. Hu et al. proved that LMNPs coated with mesoporous silica loaded with Dox showed low systemic toxicity on BALB/c mice. The H&E images of major organs showed no obvious pathological abnormalities in the heart, liver, spleen, lung, and kidney, and the parameters of hematological assessment and blood biochemistry assay were all within normal ranges (Figure 7C) [34]. Similarly, Zhu et al. proved the LMNPs coated with SiO_2_ presented negligible systemic side effects since it induces no obvious renal and hepatic toxicity and no inflammatory lesions or damage in the treatment group (Figure 7D) [35].

The mechanism of the low toxicity of LMNPs has not been well investigated. However, the degradability and excretion ability of LMNPs seems to be strongly related to it, as shown in many types of research [8,9,34,35,69,77]. Many kinds of research [8,9,77] showed that LM nanomaterials can be gradually fused and degraded when exposed to an acidic environment such as in acidic lysosomes/endosomes or acid tumor microenvironment [20]. In addition, the products of degradation are Ga^3+^, which proved to be an anti-drug resistance ion in drug-resistant cancer cells. After degradation, LMNPs could be excreted efficiently through urine and feces over time. Zhu et al. demonstrated that the cumulative excretion of Ga-LMNPs increased to be 55.7% after intravenous injection for seven days (Figure 7E) [35]. Similar results were shown in the LMNPs coated with mesoporous silica [34] and LMNPs encapsulated in polymeric [69].

## 3. Fabrication of MLMTs

The fabrication methods of MLMTs are flourishing with the fast development of LM micro/nano fabrication techniques driven by the advancement of nanoscience. Up until now, an impressive number of synthetic routes to MLMTs have been developed. According to whether the asymmetry structure of MLMTs is prepared at one procedure, the fabrication methods can be divided into two categories: One-step and two-steps. Of note, the fabrication methods determine the shape of MLMTs, which is correlated to certain propulsion mechanisms. Thus, fabrication methods are specific to certain types of propulsion mechanism. Table 3 briefly summarized the fabrication methods for MLMTs, the advantages, limitations, and correlated propulsion mechanism. The following sections will explain each method in detail.

One-step synthesis means MLMTs can be prepared in one procedure. For MILMTs on the millimeter scale, the asymmetry structure is not strictly required. Almost all the MLMTs in the millimeter scale are created by the fluidic jetting method, which is convenient and efficient to mass-produce MILMTs powered by chemical fuels. Of note, this jetting method can only generate MILMTs in spheres from the cylindrical syringe needles; thus, only chemical propulsion can be applied to motivate this type of MLMTs. For MLMTs below mm, the asymmetry structure design is required to achieve effective propulsion. Three adopted approaches for the formation of MCLMTs by directly creating asymmetry structure: Transfer printing method [49], pressure-filter-template technique [8,9,82], and ultrasound-assisted physical dispersion method [25].

**Figure 8:**
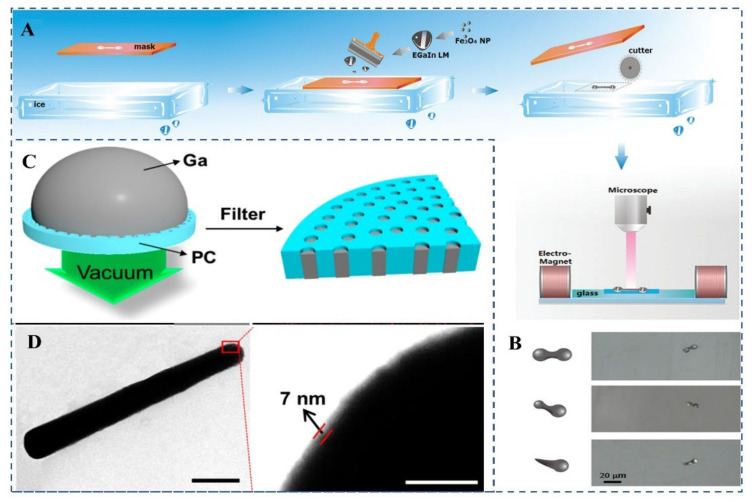
One-step fabrication methods for MLMTs. (**A**) Schematic illustration of the fabrication of MCLMTs through the ice-assisted transfer method [49]. (**B**) Microscopic images of the LM micromotor in the dumbbell shape, in the bowling-pin shape, and the tadpole shape [49]. (**C**) Schematic illustration of the fabrication of MCLMTs through the pressure-filter-template method [9]. (**D**) TEM image shows the core-shell structure of MCLMTs through this method. Scale bar, 1 μm. The enlarged TEM image indicates that a 7 mm oxide layer on the surface help stabilize the shape of motors. Scale bar, 50 nm [9].

Transfer printing is a facile technique for micro and nano-fabrication, which patterns various materials into desired functional layouts by utilizing different masks and the sacrifice layer. This technique is a universal method to prepare MNMTs in any kind of propulsion mechanism. Because theoretically, the pre-designed masks can be in the asymmetric shape of Janus spheres, rods with two ends differ in sizes, or screw-like shapes, which are essential for chemical and electrical, acoustic and light, magnetic propulsion, respectively. When applied in the fabrication of MCLMTs, however, the removal of the hard sacrifice layer easily breaks its well-designed structure due to the soft surface oxide of MCLMTs. Liu et al. proposed the ice-assisted transfer method to tackle this problem [49]. Briefly, LM is printed directly inside a mask set on an ice layer, which will melt and evaporate later; then, the direct transfer of MCLMTs to diverse substrates is finished (Figure 8A). Ice as a sacrificial layer allows the MCLMTs to stay in a pristine manner without damage or breach by any thermal stress and harsh etchants. Mask used in this method determines the shape of MCLMTs, which can be versatile such as the dumbbell shape, bowling-pin shape, and the tadpole shape (Figure 8B). However, it is worth noting that the surface properties of the ice layer highly determine the structure and morphology of MCLMTs. Therefore, a smooth ice layer with extremely low roughness and little structural discontinuities is required to give MCLMTs a well-patterned shape, which is laborious to mass-produce well-formed and homogenous MCLMTs. The pressure-filter-template technique overcomes the high surface tension of LM and utilizes oxidability to maintain the shape of soft LM motors [9]. This method requires a membrane as the template and vacuum accessories to filter the LM into the template in a controlled manner (Figure 8C). The membrane helps the shape formation of LM nanomaterials and external pressure overcomes the problem of high surface tension of LM and pore blocking. The well-designed membrane can create MCLMTs with two ends that differ in size, which is stabilized by the thin oxide (thickness ~7 nm) (Figure 8D). This method is facile to obtain MCLMTs with narrow size distribution and controlled shape. However, it requires sophisticated equipment and mass-produce is time-consuming. 

The ultrasound-assisted physical dispersion method is the most common way to fabricate LMMMs. In most cases, ultrasonication creates symmetrical LM particles. By adjusting parameters, the asymmetric rod with one end larger than another can be prepared directly, which is essential for ultrasound and light-based propelling.

The two-steps synthesis involves two procedures: The fabrication of LMMMs and the following asymmetry structure design. When the size of MLMTs decrease to micro/nanoscale, the asymmetry structure design is required. Compared to the fabrication of noble metal or inorganic materials, the mass production of LMMMs requires no expensive substrate material or complicated process. Up until now, a great number of synthesis methods for the construction of LMNPs are developed. They can be divided into two categories: Top-down and bottom-up (Table 4). The latter is too complicated so has less possibility to be applied to motors preparation. Among the bottom-up processes, the ultrasonication method is the most common one in preparing MCLMTs below mm. The fabrication methods for MCLMTs are required to be able to create micro/nanostructure with a designed shape. Thus, molding, SLICE, and blending are less possible to be applied to MCLMTs because they produce LM micro/nanostructure with either uncontrollable shape or occasionally shape defect. Besides, microfluidic flow-focusing and pulsed laser irradiation may be applied to MCLMTs in the future because they can create micro/nanostructure with a tunable and smaller size while requiring complex devices.

**Figure 9:**
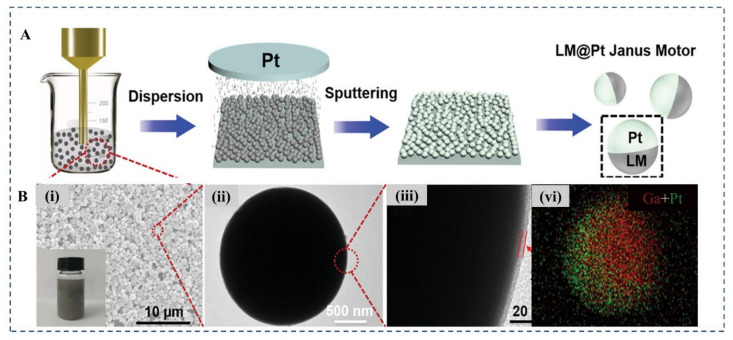
Two-steps fabrication methods for MLMTs. (**A**) Fabrication of the LM-Pt Janus particles via sputtering Pt onto LMNPs produced by ultrasonication [6]. (**B**) (**i**) SEM image of the LM spheres fabricated by ultrasonic dispersion. (**ii**) The symmetric LM sphere fabricated by ultrasonication is shown by the TEM image. (**iii**) A thin Ga_2_O_3_ shell on the LM sphere. (**vi**) The LM-Pt the distribution of the asymmetric elements after spurting is shown by EDS elemental mapping. [6].

After successfully synthesized LMNPs, the asymmetry structure design is required in the following procedures to produce efficient motion. Physical vapor deposition (PVD) including vapor-deposition or e-beam spurting are mature techniques to create asymmetry on nanomaterial. For instance, the LMNPs, produced by ultrasonication, could be easily physically deposited with metal coatings Pt to form asymmetrical Janus structure and driven by different chemical impetus (Figure 9) [6]. Other strategies, such as those modified with functional enzymes on one side of the particles, also have the same effect, which may be applied in the fabrication of MCLMTs in the future.

## 4. Propulsion Mechanism of MLMTs

Based on the abundant stimuli response properties of LM, MLMTs could be propelled by different physical energy sources. The chemical propulsion and external physical actuation have been achieved in various MLMTs. Details of motion mechanisms and performance of different types of MLMTs are summarized in Table 5.

### 4.1. Chemical Propulsion

Chemically propelled MNMTs require chemical fuels and active materials to conduct a reaction that results in autonomous propulsion. The choice of fuels and reaction depends on the properties of materials. Because of the embrittlement effect, fluidity, and high conductivity, MLMTs can be propelled by water [95], NaOH [83,96,98], H_2_O_2_ [6], and solution with PH difference or ion gradient [97].

For MLMTs propelled in water and NaOH, the driving force is mostly bubble recoil force, which arises from the addition of Al into LM to generate bubbles. The reaction is

(2) $$2\mathrm{Al}+6H_{2}O\overset{\mathrm{Ga}}{⟶}2{\mathrm{Al}(\mathrm{OH})}_{3}+3H_{2}↑$$

**Figure 10:**
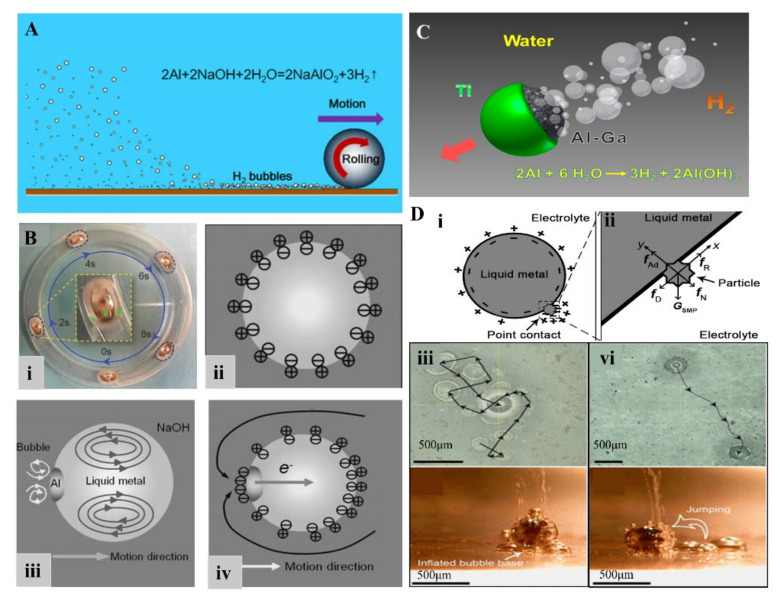
Chemically propelled MLMTs in H_2_O or NaOH. (**A**) Schematic illustration of the self-powered MLMTs [83]. (**B**) EGaIn-Al motors (**i**) Schematic for the running of EGaIn-Al motors in the circular channel. (**ii**)–(**iv**) Schematic illustration of the bipolar electrochemical mechanism [96]. (**C**) Ni-LM motors (**i**) Schematic illustration of the generation of H_2_ bubbles: point contact-induced surface charge accumulation. (**ii**) Force analysis of Ni particles contacted with LM. (**iii**) Ni-LM motors roll on a hydrophilic quartz substrate. (**vi**) Ni-LM motors jump on a hydrophobic PP substrate [98]. (**D**) Schematic illustration of water-driven hydrogen propelled MLMTs [95].

The LM alloys play a significant role in controlling the speed of Al-water reaction and production of H_2_. The dense protective oxide layer, which hinders the aluminum-water reaction, can be removed by the LM embrittlement into aluminum. Thus, the continuous hydrogen generating and continuous propulsion of MLMTs can be achieved. Based on that, Liu’s lab proposed swarms of GaIn_10-_Al motors (GAMTs) around 1 mm [83]. These MLMTs can be quickly activated in NaOH solution and performed Brownian-like random motions. It is worth mentioning that the activation of these tiny motors depends on the ingenious design of electron density distribution on LM. When GAMTs contact the bottom rough glass surface, it induced larger electron density at the bottom of GAMTs, which contributes to the reaction of Al-water (Figure 10A). Thus, a great number of bubbles occur from the bottom and propel GAMTs. The same group reported an EGaIn-Al motor can display unconventional behaviors such as autonomous convergence, divergence due to the intrinsic liquid nature of these motors [96] (Figure 10B). The research also shows that the hydrogen bubbles recoil force is not enough to power the movement of motors when the size of these MLMTs increased, and the bipolar electrochemical mechanism as additional impetus is required. Similarly, Gao et al. reported a water-driven Janus micromotor composed of Al-Ga and half-coated with Ti (Figure 10C) [95], which can move at remarkable speeds of 3 mm/s (150 body length/s). However, the coating and oxide on its surface diminish its deformation and fluidic properties. 

**Figure 11:**
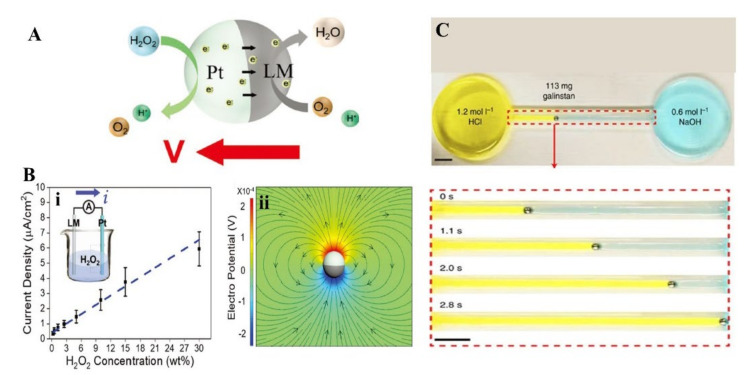
Chemically propelled MLMTs in H_2_O_2_ or solution with PH difference. (**A**) Schematic illustration of the propulsion mechanism of Pt/Galinstan Janus motor [6]. (**B**) Electrochemical measurements (**i**) the current density of the LM–Pt system and simulated results (**ii**) of the electrical potential field around Pt/Galinstan Janus motors [6]. (**C**) LM droplet swims from 1.2 mol/L HCl to 0.6 mol/L NaOH reservoir [97].

In addition to adding aluminum to LM, other particles on LM in NaOH solution can also actuate MLMTs and even achieve 3D movement. In 2016, Tang et al. reported that adding nickel particles to LMMDs (Ni-LM motors) in NaOH solution would induce LMMPs to jump or roll, which is the first time to achieve 3D movement of MLMTs [98]. This is attributed to the electron discharge effect, since the point-contact between LM and Ni particles significantly enhanced the near-surface electric field intensity at the particle tips and thus induce drastic H_2_ generation. When placed on different substrates (hydrophobic substrates or hydrophilic ones), bubbles aggregating on the substrate would induce different movements (rolling or jumping) (Figure 10D). Another chemically propelled MLMTs are Pt/Galinstan Janus sphere, moving in H_2_O_2_ (Figure 11A) [6]_._ The electrochemical measurements and simulated results confirm that the asymmetric distribution of protons creates an electric field that propels the motors (Figure 11B). Thus, the driving mechanism is self-electrophoresis [103,104]. Besides, LMMDs can also be propelled by PH or ionic concentration gradients [97]. With the experimental conditions of 1.2 mol/L HCl and 0.6 mol/L NaOH, the MLMTs traveled at a maximum velocity of 25 mm/s from the HCl side to the NaOH side (Figure 11C). This is attributed to both deformation and surface Marangoni flow. 

### 4.2. External Stimuli-Based Propulsion

#### 4.2.1. Electrical Propulsion

Among the typical external-field methods, electric fields are highly convenient for practical purposes, since they are easily accessible and do not require a complex device. Besides, their magnitudes, phases, and frequencies can be easily adjusted. The electric field can not only propel MLMTs, but also can control the speed and direction of MLMTs. The propulsion and sometimes deformation of MLMTs are also named as continuous electrowetting (CEW), which is an electrical analogy of the flow motion motivated by a thermally induced surface-tension gradient, i.e., the Marangoni effect.

**Figure 12:**
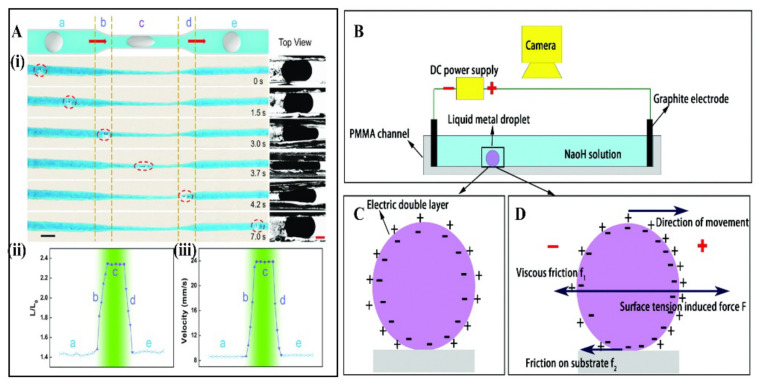
Electric-field-propelled MLMTs. (**A**) MLMTs deform and speed up in confining channels under a 30 V DC electric field. (**i**) A schematic illustration of the movement and deformation of MLMTs in a channel with different section widths. (**ii**) The relationship of the deformation degree (L/L_0_) of MLMTs and its position in the channels. (**iii**) The instantaneous velocity of MLMTs [41]. (**B**) The schematic representation of the experimental setup and the CEW effect [105]. (**C**) EDL distribution state without an external electric field [105]. (**D**) EDL distribution state under an external electric field [105].

Wang et al. reported a Galinstan micromotor moved at a speed of 117.2 mm/s after applying a DC electric field with a voltage of 30 V [41]. These MLMTs can adaptively and automatically deform and accelerate in narrow channels (Figure 12A). The mechanism behind electric field-driven MLMTs can be explained by the electrocapillary effect, which is highly related to the liquid nature and high surface tension of LM. Specifically, when a MLMTs is placed in an electrolyte (Figure 12B), an electrical double layer (EDL) appeared (Figure 12C). According to Lippmann’s equation, when a voltage is applied between the electrodes, the surface charge of the LM droplets will redistribute to reach an electrical equilibrium. Thus, the electric potential difference on the EDL will vary. This can be given by

(3) $$\gamma =\gamma_{0}-\frac{c}{2}V^{2}$$

where γ is the surface tension, c is the capacitance per unit area of the EDL, V is the electrical potential across the EDL, and $\gamma_{0}$ is the maximum value of surface tension at V = 0. 

According to Young–Laplace’s equation:

(4) $$\Delta p=p_{L}-p_{R}=\left(\gamma_{L}-\gamma_{R}\right)\frac{2}{r}=\frac{2\Delta \gamma }{r}$$

This gradient of an electric potential across the EDL produces a pressure difference. This pressure difference is the strong driving force of MLMTs (Figure 12D). It is worth noting that this method can propel LM droplets that are in a proper size (0.2 mm–1 cm), but the electrowetting may not appear if the MLMTs are small to the micron scale. When the size of MLMTs increase beyond 1 cm, the elongation of LM droplets during movement will occur.

#### 4.2.2. Acoustic Propulsion

Ultrasonic waves are widely used in medical applications and have been developed for decades [106]. As an energy source with minimal harm to the human body [107,108], ultrasound-propelled motors have the advantage of being applicable to living organisms. The ultrasound utilized in propelling motor works in a biologically safe frequency and power range [48,49]. Most of the ultrasonic power used to drive MNMTs does not exceed 740 mW/cm^2^ for diagnostic ultrasound specified by the US Food and Drug Administration (FDA) [109,110]. For example, the power density of the ultrasonic-driven nanomotor proposed by Mallouk’s team is 249.5 mW/cm^2^ [111] and (13 ± 1) mW/cm^2^ [112], and the ultrasonic drive power density of the motor prepared by Joseph’s team is 383 mW/cm^2^ [113]. 

**Figure 13:**
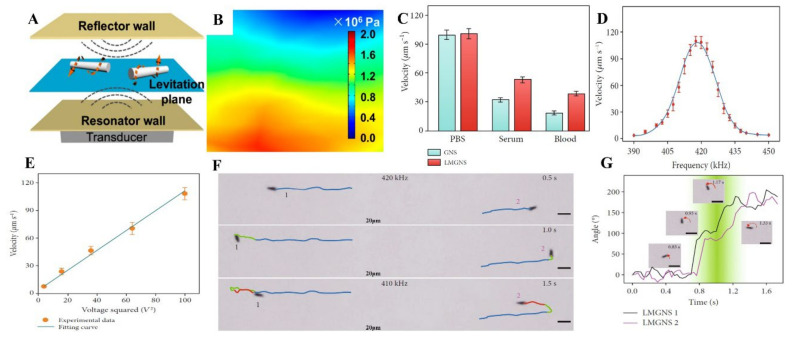
Ultrasonic propelled MLMTs. (**A**) Schematic illustration of propulsion of the asymmetric MLMTs in acoustic field. (**B**) COMSOL simulation of the generated pressure gradient along MLMTs [9]. (**C**) The time-lapse images of the direct control of MLMTs by frequency of the acoustic field. (**D**) The relationship between the velocity of MLMTs and frequency of the acoustic field. (**E**) The relationship between the velocity of MLMTs and the applied voltage of acoustic field. (**F**) The direction control of MLMTs by changing frequency. (**G**) The velocity of MLMTs in PBS, serum, and blood media [2].

The safety of material and propulsion strategies of MNMTs are dispensable in the application of biomedicine fields. To meet this demand, Wang et al. first reported ultrasound propelled MLMTs (Figure 13A) [9]. It has an asymmetrical rod shape with one larger end and another shorter end, which is essential for acoustically propelled movement. The acoustic propulsion mechanism was investigated by experiments and a finite element simulation in COMSOL Multiphysics, proving that the primary acoustic radiation force is the main driving force of MLMTs. The primary acoustic radiation force acting on the asymmetric shaped MNMTs results in the acoustic pressure difference (Figure 13B), thus achieve MNMTs propulsion [111].

Acoustic propulsion successfully solved one of the biggest challenges faced by chemically propelled MNMTs when applied in biomedicine. Salt concentration in biological media will significantly decrease the velocity of chemically driven motors. In contrast, the ultrasonic propelled MLMTs can swim at a high speed in biological media (Figure 13C). He’s group improved the velocity of MLMTs by modifying the shape of MLMTs from rod to needle and coating them with leukocyte membrane [8]. Furthermore, the velocity can be modulated on demand by regulating the frequency (Figure 13D) and voltage (Figure 13E) of the applied ultrasound field. Under the ultrasound field with 10 V and the frequency of 420 kHz, the MLMTs could reach a speed up to 108.7 μm/s. In addition, the ultrasonic frequency can not only regulate the velocity of the MLMTs but also change their motion direction inversely (Figure 13F) or in a controlled manner (Figure 13G). 

#### 4.2.3. Magnetic Propulsion

MLMTs propelled by the magnetic field have the advantages of being easily control and navigation, which are desired in many fields. Besides, the magnetic field is non-invasive and safe compared with the toxicity and the formation of byproducts of chemical fuels. The propulsion mechanism of magnetic motors depend on the type of magnetic fields and motor design. 

There are three fundamental mechanisms of MLMTs driven by magnetic fields: (a) The forces applied due to the magnetic field gradients, namely “magneto-phoretic motion”, induced by spatially inhomogeneous fields, and (b) the magnetic torque transfer, which requires rotating external, spatially homogeneous or heterogeneous magnetic fields, and (c) the Lorentz force generated due to an eddy current, induced by moving a permanent magnet.

**Figure 14:**
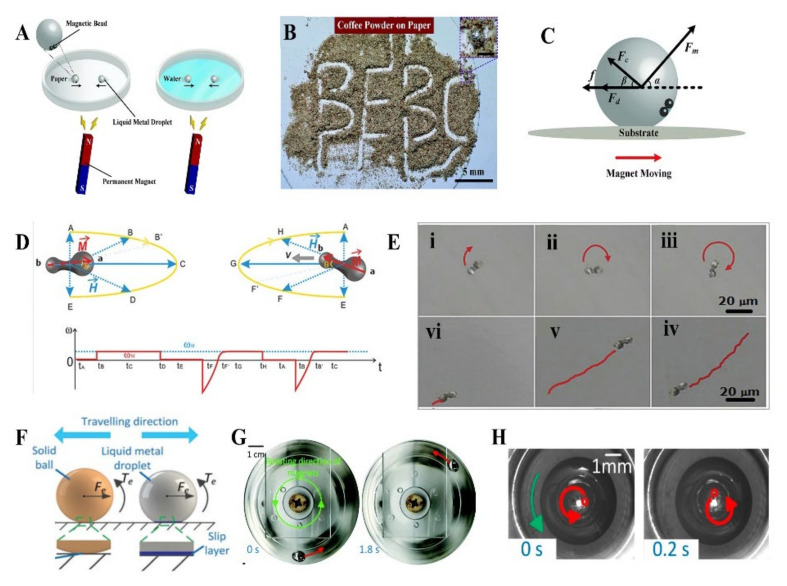
Magnetic field propelled MLMTs. (**A**) A schematic illustration of the directed magnetic controlled MLMTs. (**B**) The magnetically driven shuttling of MLMTs through coffee powder on a piece of printing paper. (**C**) Force diagram of magnetically driven MLMTs [99]. (**D**) A schematic illustration of the mechanism of the bowling-pin-like MLMTs in an elliptically polarized magnetic field. (**E**) Continuous captures of rotation of MLMTs under rotating magnetic (**i**)–(**iii**) and translational motion of MLMTs under an elliptically polarized magnetic field (**vi**)–(**iv**) [49]. (**F**) A schematic illustration of the actuation of a solid ball and a MLMTs placed on a substrate. (**G**) Continuous snapshots for the locomotion of a MLMTs [100]. (**H**) Time-lapse images of the self-rotation of MLMTs [101].

The first category of magnetically propelled MLMTs requires the incorporation of magnetic nanomaterials into LM to generate magnetic force. Due to the liquid nature of LM, it is easy to incorporate various magnetic nanomaterials such as Ni and Fe nanoparticles into the LM droplet. Liu et al. proposed magnetic actuated MLMTs in an easily accessible manner [99]. By simply encapsulating several microsized steel beads, MLMTs can operate both in water and on a solid (paper) substrate (Figure 14A). This motor can achieve highly directed motion, even finish the specific pattern of “BEBC” on a coffee powder-coated paper surface that needs accurate guided locomotion (Figure 14B). The underlying mechanism behind this magneto-phoretic motion is the balance of mechanics between the drag force and magnetic force (Figure 14C). Specifically, the total magnetic force experienced by the MLMTs can be obtained by

(5) $$F_{m}=\frac{M\Delta \chi }{\rho \mu_{0}}(\nabla B)B$$

where M is the total mass of the steel beads; ρ is the density of the steel beads; Δχ is the difference in magnetic susceptibility between the steel beads and the LM; B and ∇B are the magnetic flux density and magnetic field gradient, respectively. This shows that spatially inhomogeneous magnetic fields (∇B≠0) are indispensable. These MLMTs have been demonstrated to show great potential in the healing of paper-based flexible electronics, cargo transfer, and vessel cleaning.

The second category of magnetically propelled MLMTs requires not only magnetic nanomaterials, but also breaking the symmetry in the motors’ structure design. Special designs such as helical tails, flexible planar tails, or screw-like shapes are required. Liu et al. reported dumbbell-like Fe_3_O_4_ nanoparticles-incorporated MLMTs propelled by magnetic fields [49]. The addition of Fe_3_O_4_ nanoparticles was put. through vigorous stirring, which is a common strategy of incorporating magnetic nanoparticles [114,115,116]. The magnetic torque acting on the micromotor can be described by

(6) $${\vec{\tau }}_{M}=\vec{M}\times \vec{H}$$

where $\vec{H}$ is the magnetic field vector; $\vec{M}$ is the magnetic moments of the micromotor.

As the magnetic field rotates, the torque will act on the MLMTs, making the motor wobble rapidly. This wobbling immediately breaks the symmetry of the pressure distribution in fluid and leads to the directional motion of micromotors in a periodic way (Figure 14D). These MLMTs have “rotation” and “swimming” mode under a uniform rotating magnetic field and an elliptically polarized magnetic field, respectively (Figure 14E). By varying the frequency of the applied field, the speed of MLMTs can be readily controlled. More importantly, the MLMTs can switch rapidly between “forward” and “backward” movements by alternating the polarization of the applied magnetic field.

The third one does not require the addition of magnetic materials; just pure LM droplets can be propelled by moving a permanent magnet. Shu et al. reported an innovative method for controlling the locomotion of MLMTs using Lorentz force [100]. When a permanent magnet approaches the EGaIn droplet, the magnetic flux density B experienced by the droplet increases and induces a current j within the coils, which can be expressed as:

(7) $$j=\frac{d(B\cdot S)}{Rdt}$$

The resulting force and torque will induce the rolling of the sphere. It is worth mentioning the unconventional phenomenon that the movement direction of LM droplets is towards the traveling direction of the magnet, which is opposite to a solid sphere (Figure 14F). This attributes to a slip layer that existed between the solid (glass bottom)—liquid (LM droplets) interface, which can act as a lubricant to prevent the rolling of the droplet. Consequently, the horizontal force will induce the actuation of the EGaIn droplet towards the movement direction of the magnet, which enables an easier movement maneuver than a solid sphere (Figure 14G). Besides, the self-rotation of MLMTs induced by Lorentz force can be achieved by solely utilizing a rotating magnetic field [101]. This first achieved self-rotational motion of the droplet behaves smoothly and steadily (Figure 14H). It may enable new progress in microfluidics and microelectromechanical applications.

#### 4.2.4. Light-Based Propulsion

**Figure 15:**
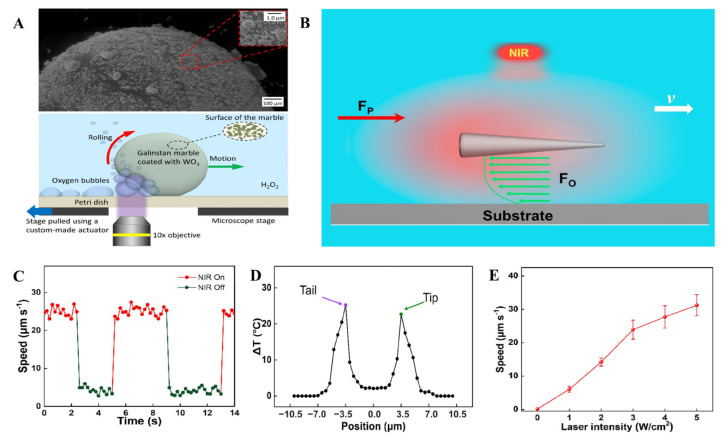
Light-driven MLMTs. (**A**) The schematic illustration of the mechanism of NIR light-driven MLMTs. (**B**) The “off/on/off” control of MLMTs. (**C**) Speed control of MLMTs by various laser power. (**D**) The temperature of the water along the long axis of MLMTs [82]. (**E**) Schematic of light-powered LM motors coated with WO_3_ particles [117].

Light-driven MLMTs have the advantages of remote maneuver on-demand with excellent spatial and temporal resolution. So far, the MLMTs have been successfully propelled by UV and NIR light. In 2013, Tang reported a UV light-driven MLMTs, which was a WO_3_ nanoparticle coated marble swimming in H_2_O_2_ (Figure 15A). With the irradiation of UV light, the semiconducting WO_3_ coating can trigger a photochemical reaction, producing O_2_ bubbles that propel the MLMTs. However, the dangers of UV light and H_2_O_2_ fuels restrict its further application in vivo. In contrast, NIR light is the most attractive stimulus among various light resources because of its minimal invasiveness and high specificity in biomedical applications. LMNPs have a high photothermal conversion efficacy under the irradiation of NIR light. Most light-driven motors require the incorporation of light-responsive materials. In contrast, MLMTs can be propelled by NIR light directly. Wang et al. reported needlelike MLMTs under the irradiation of a NIR laser (Figure 15B) [82]. These MLMTs can autonomously move at the speed of 31.22 μm/s at a laser intensity of 5 W/cm^2^. More importantly, the on-demand “start and stop” control of these micromotors can be conveniently achieved by adjusting the intensity of a NIR laser (Figure 15C). Furthermore, velocity can be effectively controlled by laser intensity (Figure 15D). The mechanism behind this light propulsion is self-thermophoresis, which is confirmed by computer simulations and experiments. Absorption of light by the LM microrod produced heat, and a local temperature gradient (~2 K across the rod) along its longitudinal axis occurred due to the difference of diameters of the two ends (Figure 15E). 

### 4.3. Hybrid Propulsion

Hybrid-driven MLMTs powered by more than one chemical or physical stimulus are attractive in situations where one of the fuels is not available or running out. More importantly, the combination of multiple driven sources enables the combination of their respective advantages. For example, the chemical propelled MLMTs cannot adjust velocity and motion direction. The electric and magnetic field can impart the adoptive motion control on chemical propelled vehicles to speed up, reverse, and other on-demand navigation. According to reports, MLMTs have been successfully propelled by the combination of chemical solution and electric fields or magnetic fields, which have more motion mode and precise motion control.

**Figure 16:**
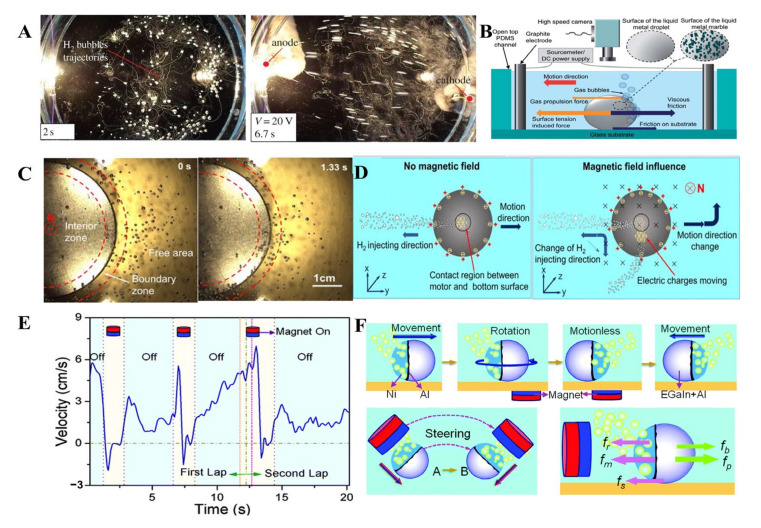
Hybrid-driven MLMTs. (**A**) Sequential snapshots of the GaIn_10_-Al motors under before (**left**) and after (**right**) applying an electric field [85]. (**B**) A schematic illustration of the forces influencing the motion of a MLMTs with or without nanoparticle coating [102]. (**C**) Continuous captures of a mass of MLMTs’ motion under magnetic field. (**D**) The schematic of electrical charge alteration is affected by the external magnetic field [86]. (**E**) The on-off motion control of the MLMTs. (**F**) The schematic of rotation and alteration of the MLMTs by a magnet [84].

Tan et al. reported a GaIn_10_-Al motor, driven by chemical solution NaOH and electric field [85]. The chemically propelled GaIn_10_ cannot control motion direction and velocity. After applying the electrical field, all the aluminum powered LM droplet would swim towards the anode very quickly like a cluster of fishes (Figure 16A), and these motors accelerate their running speed as the applied voltage increase. Similarly, Tang et al. proposed chemically driven motors coated with WO_3_ particles, the movement of which can be regulated by an electric field [102]. When applied electric field, the migration of nanoparticles on LM droplets will be altered, which triggers the change of surface tension; then, the direction of movement changed. The electric field and nanoparticle coatings can later the movement mode and direction of MLMTs, which provides an extra dimension for chemically powered MLMTs (Figure 16B). 

Tan et al. reported a novel method to restrict the motion of chemically powered motors by applying a magnetic field [86]. Through placing a permanent magnet under motors, the random motion of MLMTs will be significantly reduced and regulated into the magnetic field (Figure 16C). As discussed in Section 4.1, the glass surface at the bottom with the larger electron density tends to induce a faster reaction, and subsequently, numerous H_2_ bubbles will be emitted from the bottom and propel the motors forward. According to the Lorentz force formula,

(8) F = QvB

where Q is the value of charge, v is the speed of charge, and B is magnetic field intensity. Those electrons will be exerted by the Lorentz force; thus, the electrons congregation will be altered (Figure 16D). As a result, bubble recoil force becomes non-fixed direction, interrupting the continuous motion of motors. 

In addition, MLMTs can be propelled by the combination of three kinds of energy: Chemical solution, electric field, and magnetic field [84]. With the acceleration of the electric field, the MLMTs can run with a velocity of 3 cm/s for hours. The addition of Ni allows MLMTs to be navigated by a magnet. It can pause repetitively by switching off the magnetic field to achieve on-demand start and stop motion control (Figure 16E). Moreover, the magnet enables the MLMTs to alter the direction flexibly (Figure 16F).

## 5. Application

The unique properties of LM can endow many functions like transformation, photothermal therapy of tumor, fluidic with high conductivity, reconfigurable assembly, biosafety, degradability, and so on. Based on these properties, MLMTs have been applied in diverse fields, such as biomedicine, network repairing, assembly, and others, including cargo transfer and vessel cleaning, etc. (Table 6). It is worth mentioning that the existing application cases of MLMTs are relatively less than the abundant movement behavior and phenomena described in Section 4. Specifically, the major applications of MLMTs are on the micro/nano scale, since many applications, such as biomedicine (drug delivery in vivo), are practical when the size of machines decreases to micro/nanoscales. For MLMTs (>1 mm), the research of them mainly focuses on propulsion. Only some simple and proof-of-concept applications have been carried out. Thus, we will mainly focus on the application of MLMTs (<1 mm). If readers are interested in the applications of MLMTs (>1 mm), we recommend the corresponding literature [84,99,101]. 

### 5.1. Biomedicine

LM with softness, flexibility, biosafety, and biodegradability are attractive in biomedicine. Gao et al. proposed an Al-Ga/Ti MLMTs swimming in the water. Furthermore, the results show the speed of MLMTs was slightly affected by the biological environment [95]. However, as the MLMTs swimming in the water, Al is depleted gradually, and no navigation can be applied in these motors, which limits their further application in the human body. As described in Section 4.2.2, MLMTs propelled by ultrasound have many more advantages to be applied in biomedicine. Wang et al. proposed a MLMTs driven by ultrasound [9]. This MLMTs can actively seek cancer cells via the navigation of ultrasound and drill into cancer cells. After entering cells, it transformed from a rod to a droplet due to the removal of oxide in the acidic environment of cancer cells. These transformed MLMTs could fuse into a large droplet and then photothermally kill cancer cells under irradiation of near-infrared light. 

**Figure 17:**
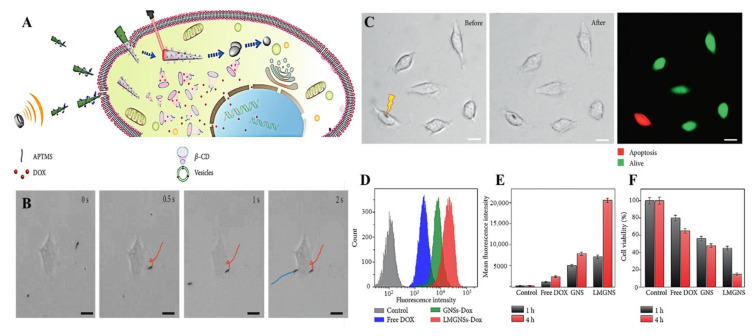
Biomedical application of MLMTs [8]. (**A**) Schematic illustration of the ultrasonically driven MLMTs coated with leukocyte membrane actively target, penetrate, and kill the cancer cell. (**B**) Time-lapse images of ultrasonically driven MLMTs actively seeking and targeting the HeLa cell. Scale bars, 20 μm. (**C**) Microscopic images of HeLa cells with the MLMTs before and after NIR laser irradiation and the fluorescence image of HeLa cells after NIR irradiation. (**D**) Quantitative Dox release analysis of the free Dox, GNS-Dox, and MLMTs-Dox treatments for 4 h by flow cytometry. (**E**) Mean fluorescence intensity of Hela cells incubated with PBS, free Dox, GNSs-Dox, and MLMTs-Dox for 1 and 4 h. (**F**) Therapeutic efficiency of free Dox, GNSs-Dox, and MLMTs-Dox to HeLa. All scale bars, 20 μm.

To further enhance the cancer therapy of MLMTs, Wang et al. reported a leukocyte membrane-coated and drug-loaded gallium MLMTs propelled by ultrasound (Figure 17A) [8]. Compared with gallium nanoswimmers without modification (GNS), the leukocyte membrane coating enables these MLMTs the function of avoiding biofouling during the motion in blood as well as cancer cell recognition. The modification on the shell of MLMTs also enables the load of Dox. These MLMTs can actively seek, penetrate, and internalize into the cancer cells (Figure 17B), after the irradiation of 808 nm laser at a power of 15 mW/μm^2^ for 5 s, a clear shape transformation was observed (Figure 17C) and the loaded drug dropped off from the transformed MLMTs. Besides, the cellular uptake (Figure 17D) and the drug release rate (Figure 17E) were enhanced by the coating of the leukocyte membrane, thus the cancer-killing rate was increased as well (Figure 17F). Thus, these ultrasound MLMTs achieved enhanced anticancer efficiency by combined photothermal and chemical therapy.

### 5.2. Network Repairing

**Figure 18:**
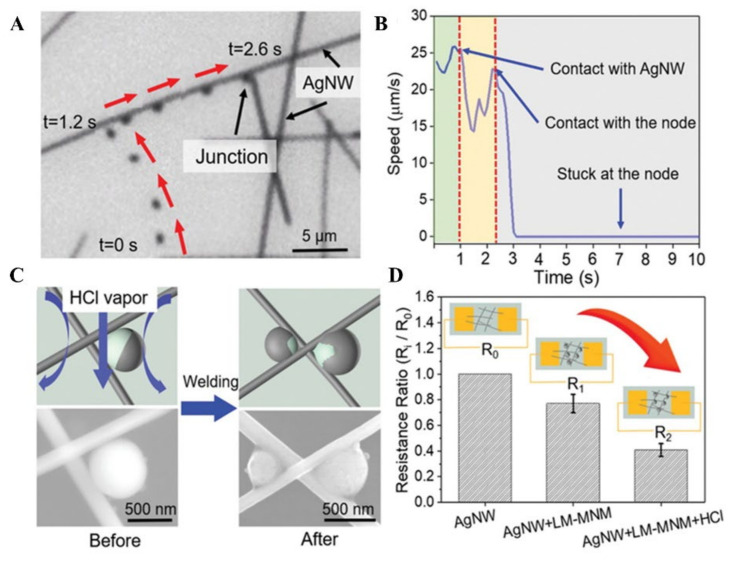
Micro welding application of MLMTs [6]. (**A**) Video snapshot and (**B**) instantaneous speed of MLMTs moving within the silver nanowire network (AgNW) network. (**C**) Schematic illustration and the corresponding SEM images of MLMTs stuck at an AgNW junction before and after HCl treatment. (**D**) The electrical resistance of a gapped gold electrode under different conditions: with AgNW network only (R_0_), and with AgNW and MLMTs before (R_1_) and after (R_2_) HCl treatment, respectively.

LM has the unique properties that it is fluidic at room temperature while maintaining the same electrical conductivity as regular metals. The fluidic properties endow the metal characteristics of soft and flexible, therefore MLMTs are easier to fit micro narrow spaces. Thus, any type of MLMTs can be utilized in network repairing, as long as the environment temperature is higher than the melting point of LMs. Based on that, Wang et al. reported a phoretic Galinstan MLMTs, which can be applied as an on-demand, self-targeting welding filler [6]. These MLMTs, half-coated with a thin layer of platinum (Pt) are capable to move in H_2_O_2_ via self-electrophoresis. After putting in H_2_O_2_ with a silver nanowire network (AgNW), they can move along the nanowires (Figure 18A) and accumulate at the contact junctions (Figure 18B). However, because of the presence of the oxide layer of Ga_2_O_3_ on the surface of MLMTs, they have a low conductive property and have small contact with the silver nanowire networks, thus unable to reduce the contact resistance significantly. By exposing MLMTs with acid vapor, the Ga_2_O_3_ layer was removed rapidly and the inner pristine LM flowed out and readily formed metallic bonds with silver at the junctions of the AgNWs (Figure 18C). Compared to the resistance of the AgNW network before and after adding MLMTs with or without acid vapor treatment, the contact resistance was shown to decrease by more than 50%, suggesting that MLMTs have a great potential to repair silver nanowire network (Figure 18D).

### 5.3. Assembly

**Figure 19:**
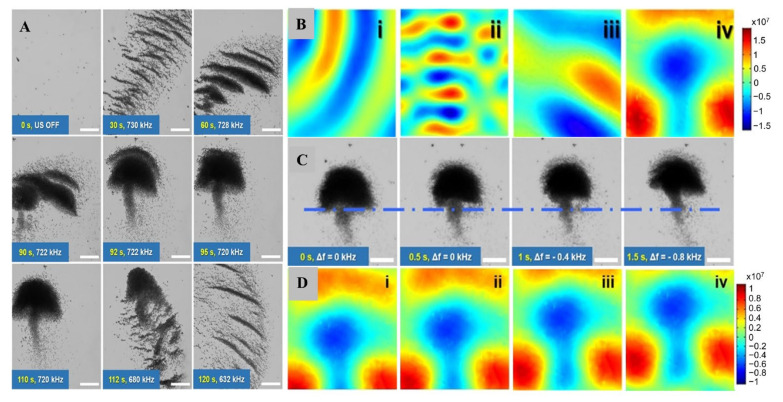
Reconfigurable assembly of ultrasonically driven MLMTs [21]. (**A**) Time-lapse images of acoustic-triggered dandelion-like assembly of EGaIn colloidal motors, including the formulation of a bud (0 to 30 s), flower bloom (60 s to 90 s), the process of fruiting (92 s to 110 s), and blowing in the wind (112 s to 120 s). Scale bars, 60 μm (0 s), 30 μm (30 s to 120 s). (**B**) Sound pressure field simulation for acoustic triggered dandelion-like assembly. The corresponding acoustic field frequencies of each simulation are: (**i**) 730 kHz, (**ii**) 728 kHz, (**iii**) 722 kHz, (**iv**) 720 kHz. (**C**) Time-lapse images of the collective motion of the EGaIn motor cluster. Scale bar, 40 μm. (**D**) Sound pressure field simulation results correspond to the experimental results as shown in (**C**). Corresponding frequency changing are: (**i**) 0, (**ii**) −0.4 kHz, (**iii**) −0.6 kHz, (**iv**) −0.8 kHz.

One of the significant features of MLMTs is the ability to perform collective self-assembly. This is extremely attractive not only because it mimics the swarming phenomenon in nature but also the complex mission in micro/nano scales cannot be performed without the adaptive and interactive functions of a group of MLMTs. LM with stimulus-responsive properties is a potential ideal smart material for producing reconfigurable active soft matter materials and systems. Li et al. reported the reconfigurable assembly of rod-shaped EGaIn MLMTs, which can mimic the dandelion flower’s growing process [25]. These MLMTs were propelled by an ultrasonic field and autonomously move at a speed of 41.2 μm/s. By modulating the frequency of the applied acoustic field, these MLMTs could self-organized into various patterns that mimic the flower’s growing process including the formulation of a bud, flower bloom, the process of fruiting, and blowing in the wind (Figure 19A). The mechanism behind this assembled process was investigated by numerical simulation, which revealed that the acoustic propulsion in combination with steric repulsion and hydrodynamics were synergistically performed the dynamic pattern formation of EGaIn dandelion flower-like clusters (Figure 19B). In addition, the assembled dandelion flower-like MLMTs can collectively move by adjusting the frequency of the applied acoustic field (Figure 19C). The mechanism behind this collective movement is that the change of frequency of the acoustic field leads to the variation of the node planes, thus driving the cluster to move (Figure 19D). It is worth mentioning that it is relatively easier to facilitate the aggregation of MNMTs by ultrasound, because there are ultrasonic nodes in the ultrasonic field. Although some similar phenomena of the other MLMTs were reported before, the controllable and dynamic swarms which mimic the biological phenomena was first proposed. The question of whether LM materials affect pattern formation still remains, and the mechanism of the interaction between MLMTs and ultrasound should be further investigated. 

## 6. Future Outlook

As surveyed above, LM has unique properties that can endow MNMTs with novel properties and functions. Furthermore, it has achieved chemical, magnetic, optical, ultrasonic, and hybrid propulsion. However, there are still many challenges that need to be addressed urgently.

### 6.1. Fabrication

Although considerable efforts have paid to great progress in the diversity of MLMTs, more works need to be attributed to the fabrication of MLMTs to promote the development of MLMTs since it significantly influences the propulsion and function of motors. First, facile and practical approaches should be developed to achieve the mass-produce of MLMTs. Although the recent flourishing of fabrication techniques of LMNPs shows more research have paid attention to this area, only several of them can meet the core requirement of MLMTs fabrication: The controlled and well-designed asymmetric shape. Many LMNPs fabrication methods focus on the important aspects: Further decrease of size or enhanced stability, while it is also a promising perspective to work on how to create LM nanomaterial with controlled asymmetric shape, which is better to have the merits of lower cost, high efficiency, and small size distribution. Second, the functionalization of MLMTs needs further investigation to expand the scope of potential applications. The addition of other materials and the surface modification of LM nanomaterials, which endows MLMTs more functions, need to be widely investigated as that of bulk LM materials. 

### 6.2. Propulsion

Chemical, electric, magnetic, optical, ultrasonic, and hybrid propulsion of MLMTs have been achieved while other fuels (such as an enzyme) and their combination are worth applying on MLMTs to achieve more precise maneuver. So far, the most common strategy to control the direction of MLMTs is incorporating magnetic materials and applying magnetic fields. Although magnetically propelled MLMTs can be steered by a magnetic field and achieve forward and back motion, the low solubility of magnetic materials in LM restricts its more precise motion control, and the magnetocaloric effect shortens the lifetime of as-prepared MLMTs. Thus, precise direction control needs further improvement. In addition, driving force and speed need to be further improved. As described in a recent review [118], a strong driving force is essential in the cell membrane opening, which is one of the key parameters in the biomedicine application of MLMTs. However, the existing LMNPs and MLMTs enter cells by endocytosis, which requires a long time (normally 24 h) and has low efficiency [9,119]. Thus, it will significantly enhance the efficiency of cargo delivery if the driving force enhancement and cell membrane opening is efficiently achieved by MLMTs, which can be a milestone in biomedicine application [118]. 

### 6.3. Applications

LM, as a group of new and unique materials, can cover the shortage and supplement the vacant applications of conventional rigid materials. For example, the combination of liquid nature and high electrical conductivity of LM makes it a suitable candidate for nanonetwork repairing, which is hard to be achieved by conventional rigid metals. However, the existing application cases of MLMTs are less than the abundant propulsion research; thus, the combination and innovation of MLMTs and the extensive applications of LMs need more exploration and investigation. First, the shape-transformation properties of LM have already endowed the function of endosome-escape, biofilm disruption, and will play more roles in many potential fields including infection control, biofilm removal, smart-bandage materials, surface sterilization, and the in-situ removal of biofilms from medical apparatus [22]. If MLMTs with controllable movement can achieve these tasks, the efficiency and effectiveness can be significantly enhanced. Besides, the stimulus-responsive properties of LMs have much potential in the dynamic swarms and reconfigurable assembly, which are not only an intriguing phenomenon but also fundamental for group cooperation of MNMTs to achieve the complex mission in microscale worlds [120]. Moreover, LMs are ideal materials for a wide potential application in biomedicine like cancer therapy [31,121], embolism [10], and the rewarming agent in organ cryopreservation [119]. Compared with the static LMNPs, MLMTs with movement may solve many existing challenges in these fields by actively targeting cancer cells, assembling where embolization is needed, and entering cells by opening cell membranes. To achieve these envisioned applications, there are still some challenges to be resolved: Improving the velocity and lifetime of MLMTs, reversible fusion and fission, controllable group interaction, and so on. It is expected that the association of LMs with motors will bring new and unique opportunities to the field of micro/nanomachines. 

## 7. Conclusions

In summary, the properties of LM and the progress of MLMTs and future challenges have been systematically reviewed for recently emerging MLMTs. LM with unique properties of liquid nature and metallic properties will endow MLMTs’ new properties and functions. LMs are shaped transformable which can fuse into droplets and degrade in a cell, and have the function of disrupting the endosomal membrane to achieve drug delivery and bacterial biofilm membrane to kill bacterial. The liquid nature and high electricity enable MLMTs to actively repairing the nano-Ag network and significantly lower its contact resistance. In terms of optical properties, functional LMPs with NIR photothermal effects and loaded with drugs have achieved great inhibition of tumor growth by the combination of photothermal and chemotherapy. Besides, LM nanomaterials that are stimulus-responsive by acid, magnetic, and temperature can have a multi-functional application in cancer therapy, biofilm removal, and fluid mechanics. In addition, the increasing research on the biological toxicity of LM nanomaterials in vitro and in vivo shows that its biosafety and biodegradability have enormous advantages in the biomedicine field.

As for the development of MLMTs, the MLMTs can be fabricated by pressure-filter-template method, transfer printing, and the combination of ultrasonication and physical vapor deposition. With asymmetric shapes, MLMTs have been successfully propelled by chemical fuels, external energy fields and hybrid sources with motion control. It shows the good function in cancer therapy, nano network repairing, reconfigurable assembly, and other areas. Overall, MLMTs will play an increasing role in many applications and will provide a fertile ground for future discoveries and developments.

## Figures and Tables

**Table 1:** Ga-based liquid metal (LM) properties and applications.

Properties		Application	Refs.
Basic metallic characteristics	High thermal conductivity	Thermal interface materials	[12,13]
	Good electrical conductivity	Electronics	[14]
	Electromagnetic properties	Electromagnetic shielding material	[15,16,17]
	Radiopacity	Radiocontrast agent	[7,11,18]
Amorphous properties	Superb fluidity	Microfluidics	[4,5]
	Excellent flexibility	Stretchable and soft electronics	[19]
	Shape transformability	Cancer therapy	[8,9,20,21,22]
	Self-healing capability	Self-healing e-skin systems	[23]
	Reconfigurability	Soft robotics	[24,25]
	Facile functionalization accessibility	Metal composite	[26,27]
Featured properties	Biocompatibility and biodegradability	Biomedical applications	[28]
	Catalytic properties	Catalyst	[29,30]
	Photo-thermal/photodynamic capability	Cancer therapy	[31,32,33,34,35,36]
	Stimuli responsiveness	Robotics	[37]

**Table 2:** Overview of different propulsion methods of mini/micro/nano scale liquid metal motors (MLMTs).

-	-	Key Features	Pros	Cons
Chemical propulsion	In water	MLMTs are powered by bubbles generated from the chemical reactions of LM with water.	High speed good biocompatibility low cost	Lack of directional motion short lifetime
	In NaOH	MLMTs are powered by bubbles generated from the chemical reactions of LM with NaOH.	High speed low cost	Poor biocompatibility Lack of directional motion short lifetime
	In H_2_O_2_	MLMTs are powered by electrophoresis due to the electron transfer between LM and other metals which are capable of catalyzing the decomposition of H_2_O_2_.	Low cost	Poor biocompatibility Lack of directional motion short lifetime
External stimuli-based propulsion	Electrical propulsion	MLMTs are powered by an external electrical field.	Precise motion control long lifetime	Poor biocompatibility require special experimental setup
	Acoustic propulsion	MLMTs are powered by an external high-frequency acoustic (or ultrasonic) field.	Good biocompatibility non-invasive precise motion control.	The requirement for special and complex experimental devices to control the motion
	Magnetic propulsion	MLMTs are powered by an external magnetic field.		
	Light-based propulsion	MLMTs are powered by an external light source.		
Hybrid propulsion		Combination of various propulsion mechanisms to propel motors.	Multi-stimuli responsive capability	(depends on the methods)

**Table 3:** Fabrication methods for MLMTs.

Method		Pros	Cons	Propulsion Mechanism	Refs.
One-step	Pressure-filter-template method	Tunable shape and size; narrow size distribution	Complex equipment; time-consuming; hard to mass-produce	Universal	[8,9,82]
	Ultrasound-assisted physical dispersion method	Facile operation; easy to mass-produce	Wide size distribution	Acoustic or light-based propulsion	[25]
	Ice-assisted transfer printing method	Tunable shape and size	Shape defect; requirement of a smooth surface of the ice	Universal	[49]
	Fluidic jetting	Low cost; facile operation	Wide size distribution; uncontrollable size; relatively large	Chemical propulsion	[83,84,85,86]
Two-steps	Ultrasound-assisted physical dispersion method + sputtering	Facile operation; easy to mass-produce	Complex equipment	Chemical propulsion	[6]

**Table 4:** Fabrication methods for LMMMs.

Method			Size Range	Prons	Cons	Refs.
Top-down	Fluidic jetting		100 μm-few mm	Low cost; Facile operation	Wide size distribution; uncontrollable size	[83,84,85,86]
	Molding		100–3500 μm	Facile operation	Time-consuming; shape defect	[87]
	Microfluidic flow-focusing		50–200 μm	Tunable size	Complex equipment	[88,89]
	SLICE		6 nm–10 μm	Facile operation; Tunable size	Uncontrollable size and shape	[59]
	Ultrasonication		10 nm–5 μm	Facile operation	Complex equipment	[47,48,90,91]
	Pulsed laser irradiation	on solid	>200 nm	Smaller size	Complex equipment	[92]
		in liquid	~5 nm	Smallest size so far	Complex equipment	[71]
Bottom-up	Physical Vapor Deposition		10 nm–300 nm	Narrow size distribution	Smaller size	[93]
	Thermal Decomposition Method		10–30 nm	Hard operation	Smaller size	[94]

**Table 5:** Propulsion mechanism and performance of different types of MLMTs.

Category		Materials	Size	Shape	Swimming Style	Velocity	Propulsion Force	Refs.
Chemical propulsion	Water	Ga + Al + Pt	20 μm	Janus sphere	translational motion	3 mm/s	Bubbles recoil force	[95]
	NaOH	GaIn_10_ + Al	0.9–1.2 mm	sphere	translational motion + rotation	3.9 cm/s	bubbles recoil force	[83]
	NaOH	EGaIn + Al	~5 mm	sphere	translational motion	25 cm/s	Bubble + surface tension	[96]
	PH difference	Galinstan	3 mm	sphere	translational motion	100 mm/s	surface tension	[97]
	NaOH + Ni	EGaIn + Ni	240 μm	sphere	translational motion	1400 μm/s	bubbles recoil force	[98]
	H_2_O_2_	Galinstan + Pt	500–800 nm	Janus sphere	translational motion	30 μm/s	self-electrophoresis	[6]
External stimuli-based propulsion	Acoustic propulsion	Ga	5.5 μm	rod	translational motion + rotation	23 μm/s	primary acoustic radiation force	[9]
		EGaIn	850 nm	rod	translational motion + assembly	41.2 μm/s	primary acoustic radiation force	[25]
		Ga	7 μm	needle	translational motion + rotation	108.7 μm/s	primary acoustic radiation force	[8]
External stimuli-based propulsion	Magnetic propulsion	EGaIn + carbon steel beads	~2 mm	sphere	translational motion	70 mm/s	magnetic force	[99]
		EGaIn + Fe_3_O_4_	20 μm	dumbbell	translational motion + rotation	60 μm/s	magnetic torque	[49]
		EGaIn	<5 mm	sphere	translational motion + rotation	100 mm/s	Lorentz force	[100]
		EGaIn	<5 mm	sphere	rotation	100 RPM	Lorentz force	[101]
	Electrical propulsionLight based propulsion	Galinstan	2.5 mm	sphere	translational motion	117.2 mm/s	bubbles recoil force and surface tension	[41]
		Ga	7 μm	needle	translational motion	31.22 μm/s	self-thermophoresis	[82]
		Galinstan + WO_3_	1–3 mm	sphere	translational motion	70 μm/s	UV light	[102]
Hybrid propulsion	NaOH+ electric field	Galinstan	0.2–3 mm	sphere	translational motion	100 mm/s	Bubble + surface tension	[102]
		GaIn_10_ + Al	2 mm	sphere	translational motion + rotation	43 cm/s	bubble + surface tension	[85]
	NaOH+ magetic field + electrical field	GaIn_10_ + Al	<1 mm	sphere	translational motion	5.8 cm/s	Bubble + Lorentz force	[86]
		EGaIn + Al + Ni	~2 mm	sphere	translational motion + rotation	8 cm/s	Bubble + magnetic force	[84]

**Table 6:** The application of MLMTs.

Application		Category	Refs.
Biomedicine		Chemical propulsion (in water)	[95]
		Acoustic propulsion	[8,9]
Network Repairing		Chemical propulsion	[6]
Assembly		Acoustic propulsion	[25]
Others	Cargo transfer/vessel cleaning	Magnetic propulsion	[84,99]
	Cooling system/Mixer	Magnetic propulsion	[101]

## References

[1] Abdelmohsen L., Peng F., Tu Y., Wilson D.A. (2014). Micro- and nano-motors for biomedical applications. J. Mater. Chem. B.

[2] Gao W., Wang J. (2014). The environmental impact of micro/nanomachines: A review. ACS Nano.

[3] Sanchez S., Soler L., Katuri J. (2015). Chemically powered micro- and nanomotors. Angew. Chem. Int. Ed. Engl..

[4] Khoshmanesh K., Tang S.Y., Zhu J.Y., Schaefer S., Mitchell A., Kalantar-Zadeh K., Dickey M.D. (2017). Liquid metal enabled microfluidics. Lab Chip.

[5] Tang S.Y., Lin Y., Joshipura I.D., Khoshmanesh K., Dickey M.D. (2015). Steering liquid metal flow in microchannels using low voltages. Lab Chip.

[6] Wang Y., Duan W., Zhou C., Liu Q., Gu J., Ye H., Li M., Wang W., Ma X. (2019). Phoretic Liquid Metal Micro/Nanomotors as Intelligent Filler for Targeted Microwelding. Adv. Mater..

[7] Wang Q., Yu Y., Pan K., Liu J. (2014). Liquid metal angiography for mega contrast X-ray visualization of vascular network in reconstructing in-vitro organ anatomy. IEEE Trans. Biomed. Eng..

[8] Wang D., Gao C., Zhou C., Lin Z., He Q. (2020). Leukocyte Membrane-Coated Liquid Metal Nanoswimmers for Actively Targeted Delivery and Synergistic Chemophotothermal Therapy. Research.

[9] Wang D., Gao C., Wang W., Sun M., Guo B., Xie H., He Q. (2018). Shape-Transformable, Fusible Rodlike Swimming Liquid Metal Nanomachine. ACS Nano.

[10] Kim D., Hwang J., Choi Y., Kwon Y., Jang J., Yoon S., Choi J. (2019). Effective Delivery of Anti-Cancer Drug Molecules with Shape Transforming Liquid Metal Particles. Cancers.

[11] Fan L., Duan M., Xie Z., Pan K., Wang X., Sun X., Wang Q., Rao W., Liu J. (2020). Injectable and Radiopaque Liquid Metal/Calcium Alginate Hydrogels for Endovascular Embolization and Tumor Embolotherapy. Small.

[12] Chen S., Deng Z., Liu J. (2020). High performance liquid metal thermal interface materials. Nanotechnology.

[13] Mei S.F., Gao Y.X., Deng Z.S., Liu J. (2014). Thermally Conductive and Highly Electrically Resistive Grease Through Homogeneously Dispersing Liquid Metal Droplets Inside Methyl Silicone Oil. J. Electr. Packag..

[14] Wang C., Wang C., Huang Z., Xu S. (2018). Materials and Structures toward Soft Electronics. Adv. Mater..

[15] Zhang M.K., Zhang P.J., Zhang C.L., Wang Y.S., Chang H., Rao W. (2020). Porous and anisotropic liquid metal composites with tunable reflection ratio for low -temperature electromagnetic interference shielding. Appl. Mater. Today.

[16] Zhang M.K., Zhang P.J., Wang Q., Li L., Dong S.J., Liu J., Rao W. (2019). Stretchable liquid metal electromagnetic interference shielding coating materials with superior effectiveness. J. Mater. Chem. C.

[17] Yu D., Liao Y., Song Y., Wang S., Wan H., Zeng Y., Yin T., Yang W., He Z. (2020). A Super-Stretchable Liquid Metal Foamed Elastomer for Tunable Control of Electromagnetic Waves and Thermal Transport. Adv. Sci..

[18] Sun X., Cui B., Yuan B., Wang X., Fan L., Yu D., He Z., Sheng L., Liu J., Lu J. (2020). Liquid Metal Microparticles Phase Change Medicated Mechanical Destruction for Enhanced Tumor Cryoablation and Dual-Mode Imaging. Adv. Funct. Mater..

[19] Dickey M.D. (2017). Stretchable and Soft Electronics using Liquid Metals. Adv. Mater..

[20] Lu Y., Lin Y., Chen Z., Hu Q., Liu Y., Yu S., Gao W., Dickey M.D., Gu Z. (2017). Enhanced Endosomal Escape by Light-Fueled Liquid-Metal Transformer. Nano Lett..

[21] Gan T., Shang W., Handschuh-Wang S., Zhou X. (2019). Light-Induced Shape Morphing of Liquid Metal Nanodroplets Enabled by Polydopamine Coating. Small.

[22] Elbourne A., Cheeseman S., Atkin P., Truong N.P., Syed N., Zavabeti A., Mohiuddin M., Esrafilzadeh D., Cozzolino D., McConville C.F. (2020). Antibacterial Liquid Metals: Biofilm Treatment via Magnetic Activation. ACS Nano.

[23] Yang J., Cheng W.L., Kalantar-Zadeh K. (2019). Electronic Skins Based on Liquid Metals. Proc. IEEE.

[24] Wang X., Guo R., Liu J. (2018). Liquid Metal Based Soft Robotics: Materials, Designs, and Applications. Adv. Mater. Technol..

[25] Li Z.S., Zhang H.Y., Wang D.L., Gao C.Y., Sun M.M., Wu Z.G., He Q. (2020). Reconfigurable Assembly of Active Liquid Metal Colloidal Cluster. Angew. Chem. Int. Ed..

[26] Chen S., Wang H.-Z., Zhao R.-Q., Rao W., Liu J. (2020). Liquid Metal Composites. Matter.

[27] Malakooti M.H., Bockstaller M.R., Matyjaszewski K., Majidi C. (2020). Liquid metal nanocomposites. Nanoscale Adv..

[28] Yan J., Lu Y., Chen G., Yang M., Gu Z. (2018). Advances in liquid metals for biomedical applications. Chem. Soc. Rev..

[29] Liang S.T., Wang H.Z., Liu J. (2018). Progress, Mechanisms and Applications of Liquid-Metal Catalyst Systems. Chem. Eur. J..

[30] Allioux F.-M., Merhebi S., Ghasemian M.B., Tang J., Merenda A., Abbasi R., Mayyas M., Daeneke T., O’Mullane A.P., Daiyan R. (2020). Bi–Sn Catalytic Foam Governed by Nanometallurgy of Liquid Metals. Nano Lett..

[31] Sun X., Sun M., Liu M., Yuan B., Gao W., Rao W., Liu J. (2019). Shape tunable gallium nanorods mediated tumor enhanced ablation through near-infrared photothermal therapy. Nanoscale.

[32] Fan L.L., Sun X.Y., Wang X.L., Wang H.Z., Liu J. (2019). NIR laser-responsive liquid metal-loaded polymeric hydrogels for controlled release of doxorubicin. RSC Adv..

[33] Fan L., Duan M., Sun X., Wang H., Liu J. (2020). Injectable Liquid Metal- and Methotrexate-Loaded Microsphere for Cancer Chemophotothermal Synergistic Therapy. ACS Appl. Bio Mater..

[34] Hu J.J., Liu M.D., Chen Y., Gao F., Peng S.Y., Xie B.R., Li C.X., Zeng X., Zhang X.Z. (2019). Immobilized liquid metal nanoparticles with improved stability and photothermal performance for combinational therapy of tumor. Biomaterials.

[35] Zhu P., Gao S., Lin H., Lu X., Yang B., Zhang L., Chen Y., Shi J. (2019). Inorganic Nanoshell-Stabilized Liquid Metal for Targeted Photonanomedicine in NIR-II Biowindow. Nano Lett..

[36] Kulkarni S., Pandey A., Mutalik S. (2020). Liquid metal based theranostic nanoplatforms: Application in cancer therapy, imaging and biosensing. Nanomedicine.

[37] Ren L., Xu X., Du Y., Kalantar-Zadeh K., Dou S.X. (2020). Liquid metals and their hybrids as stimulus–responsive smart materials. Mater. Today.

[38] Chen S., Yang X., Cui Y., Liu J. (2018). Self-Growing and Serpentine Locomotion of Liquid Metal Induced by Copper Ions. ACS Appl. Mater. Interfaces.

[39] Chen S., Wang L., Zhang Q.L., Liu J. (2018). Liquid metal fractals induced by synergistic oxidation. Sci. Bull..

[40] Wang W., Duan W.T., Ahmed S., Mallouk T.E., Sen A. (2013). Small power: Autonomous nano- and micromotors propelled by self-generated gradients. Nano Today.

[41] Wang D., Lin Z., Zhou C., Gao C., He Q. (2019). Liquid Metal Gallium Micromachines Speed Up in Confining Channels. Adv. Intell. Syst..

[42] Liu Y., Zhang W., Wang H. (2021). Synthesis and application of core–shell liquid metal particles: A perspective of surface engineering. Mater. Horiz..

[43] Li H., Qiao R., Davis T.P., Tang S.-Y. (2020). Biomedical Applications of Liquid Metal Nanoparticles: A Critical Review. Biosensors.

[44] Daeneke T., Khoshmanesh K., Mahmood N., de Castro I.A., Esrafilzadeh D., Barrow S.J., Dickey M.D., Kalantar-Zadeh K. (2018). Liquid metals: Fundamentals and applications in chemistry. Chem. Soc. Rev..

[45] Hohman J.N., Kim M., Wadsworth G.A., Bednar H.R., Jiang J., LeThai M.A., Weiss P.S. (2011). Directing substrate morphology via self-assembly: Ligand-mediated scission of gallium-indium microspheres to the nanoscale. Nano Lett..

[46] Kumar V.B., Gedanken A., Kimmel G., Porat Z. (2014). Ultrasonic cavitation of molten gallium: Formation of micro- and nano-spheres. Ultrason. Sonochem..

[47] Yan J.J., Zhang X.D., Liu Y., Ye Y.Q., Yu J.C., Chen Q., Wang J.Q., Zhang Y.Q., Hu Q.Y., Kang Y. (2019). Shape-controlled synthesis of liquid metal nanodroplets for photothermal therapy. Nano Res..

[48] Yamaguchi A., Mashima Y., Iyoda T. (2015). Reversible Size Control of Liquid-Metal Nanoparticles under Ultrasonication. Angew. Chem. Int. Ed. Engl..

[49] Liu M., Wang Y., Kuai Y., Cong J., Xu Y., Piao H.G., Pan L., Liu Y. (2019). Magnetically Powered Shape-Transformable Liquid Metal Micromotors. Small.

[50] Kumar V.B., Gedanken A., Porat Z. (2015). Facile synthesis of gallium oxide hydroxide by ultrasonic irradiation of molten gallium in water. Ultrason. Sonochem..

[51] Lin Y., Liu Y., Genzer J., Dickey M.D. (2017). Shape-transformable liquid metal nanoparticles in aqueous solution. Chem. Sci..

[52] Farrell Z.J., Tabor C. (2018). Control of Gallium Oxide Growth on Liquid Metal Eutectic Gallium/Indium Nanoparticles via Thiolation. Langmuir.

[53] Wang H., Pumera M. (2015). Fabrication of Micro/Nanoscale Motors. Chem. Rev..

[54] Xu T., Gao W., Xu L.P., Zhang X., Wang S. (2017). Fuel-Free Synthetic Micro-/Nanomachines. Adv. Mater..

[55] Ghigna P., Spinolo G., Parravicini G.B., Stella A., Migliori A., Kofman R. (2007). Metallic versus covalent bonding: Ga nanoparticles as a case study. J. Am. Chem. Soc..

[56] Tang L., Cheng S., Zhang L., Mi H., Mou L., Yang S., Huang Z., Shi X., Jiang X. (2018). Printable Metal-Polymer Conductors for Highly Stretchable Bio-Devices. iScience.

[57] Boley J.W., White E.L., Kramer R.K. (2015). Mechanically sintered gallium-indium nanoparticles. Adv. Mater..

[58] Kim D., Thissen P., Viner G., Lee D.W., Choi W., Chabal Y.J., Lee J.B. (2013). Recovery of nonwetting characteristics by surface modification of gallium-based liquid metal droplets using hydrochloric acid vapor. ACS Appl. Mater. Interfaces.

[59] Çınar S., Tevis I.D., Chen J., Thuo M. (2016). Mechanical Fracturing of Core-Shell Undercooled Metal Particles for Heat-Free Soldering. Sci. Rep..

[60] Kumar V.B., Porat Z., Gedanken A. (2015). DSC measurements of the thermal properties of gallium particles in the micron and sub-micron sizes, obtained by sonication of molten gallium. J. Therm. Anal. Calorim..

[61] Song H., Kim T., Kang S., Jin H., Lee K., Yoon H.J. (2020). Ga-Based Liquid Metal Micro/Nanoparticles: Recent Advances and Applications. Small.

[62] Yang Y., Callahan J.M., Kim T.H., Brown A.S., Everitt H.O. (2013). Ultraviolet nanoplasmonics: A demonstration of surface-enhanced Raman spectroscopy, fluorescence, and photodegradation using gallium nanoparticles. Nano Lett..

[63] Yang Y., Akozbek N., Kim T.-H., Sanz J.M., Moreno F., Losurdo M., Brown A.S., Everitt H.O. (2014). Ultraviolet–Visible Plasmonic Properties of Gallium Nanoparticles Investigated by Variable-Angle Spectroscopic and Mueller Matrix Ellipsometry. ACS Photonics.

[64] Knight M.W., Coenen T., Yang Y., Brenny B.J., Losurdo M., Brown A.S., Everitt H.O., Polman A. (2015). Gallium plasmonics: Deep subwavelength spectroscopic imaging of single and interacting gallium nanoparticles. ACS Nano.

[65] Reineck P., Lin Y., Gibson B.C., Dickey M.D., Greentree A.D., Maksymov I.S. (2019). UV plasmonic properties of colloidal liquid-metal eutectic gallium-indium alloy nanoparticles. Sci. Rep..

[66] Catalan-Gomez S., Redondo-Cubero A., Palomares F.J., Nucciarelli F., Pau J.L. (2017). Tunable plasmonic resonance of gallium nanoparticles by thermal oxidation at low temperaturas. Nanotechnology.

[67] Vivekchand S.R., Engel C.J., Lubin S.M., Blaber M.G., Zhou W., Suh J.Y., Schatz G.C., Odom T.W. (2012). Liquid plasmonics: Manipulating surface plasmon polaritons via phase transitions. Nano Lett..

[68] Zhang W., Naidu B.S., Ou J.Z., O’Mullane A.P., Chrimes A.F., Carey B.J., Wang Y.C., Tang S.Y., Sivan V., Mitchell A. (2015). Liquid Metal/Metal Oxide Frameworks with Incorporated Ga2O3 for Photocatalysis. ACS Appl. Mater. Interfaces.

[69] Chechetka S.A., Yu Y., Zhen X., Pramanik M., Pu K., Miyako E. (2017). Light-driven liquid metal nanotransformers for biomedical theranostics. Nat. Commun..

[70] Finkenauer L.R., Lu Q., Hakem I.F., Majidi C., Bockstaller M.R. (2017). Analysis of the Efficiency of Surfactant-Mediated Stabilization Reactions of EGaIn Nanodroplets. Langmuir.

[71] Yu H., Zhao W., Ren L., Wang H., Guo P., Yang X., Ye Q., Shchukin D., Du Y., Dou S. (2020). Laser-Generated Supranano Liquid Metal as Efficient Electron Mediator in Hybrid Perovskite Solar Cells. Adv. Mater..

[72] Syed N., Zavabeti A., Mohiuddin M., Zhang B.Y., Wang Y.C., Datta R.S., Atkin P., Carey B.J., Tan C., van Embden J. (2017). Sonication-Assisted Synthesis of Gallium Oxide Suspensions Featuring Trap State Absorption: Test of Photochemistry. Adv. Funct. Mater..

[73] Zhang W., Ou J.Z., Tang S.Y., Sivan V., Yao D.D., Latham K., Khoshmanesh K., Mitchell A., O’Mullane A.P., Kalantar-zadeh K. (2014). Liquid Metal/Metal Oxide Frameworks. Adv. Funct. Mater..

[74] Love J.C., Estroff L.A., Kriebel J.K., Nuzzo R.G., Whitesides G.M. (2005). Self-assembled monolayers of thiolates on metals as a form of nanotechnology. Chem. Rev..

[75] Knop K., Hoogenboom R., Fischer D., Schubert U.S. (2010). Poly(ethylene glycol) in drug delivery: Pros and cons as well as potential alternatives. Angew. Chem. Int. Ed. Engl..

[76] Sivan V., Tang S.-Y., O’Mullane A.P., Petersen P., Eshtiaghi N., Kalantar-zadeh K., Mitchell A. (2013). Liquid Metal Marbles. Adv. Funct. Mater..

[77] Lu Y., Hu Q., Lin Y., Pacardo D.B., Wang C., Sun W., Ligler F.S., Dickey M.D., Gu Z. (2015). Transformable liquid-metal nanomedicine. Nat. Commun..

[78] Zhang M.K., Yao S.Y., Rao W., Liu J. (2019). Transformable soft liquid metal micro/nanomaterials. Mater. Sci. Eng. R Rep..

[79] Sun X., Guo R., Yuan B., Chen S., Wang H., Dou M., Liu J., He Z.Z. (2020). Low-Temperature Triggered Shape Transformation of Liquid Metal Microdroplets. ACS Appl. Mater. Interfaces.

[80] Gook D.A., Edgar D., Stern C. (2004). Cryopreservation of human ovarian tissue. Eur. J. Obstet. Gynecol. Reprod. Biol..

[81] Soto E.R., O’Connell O., Dikengil F., Peters P.J., Clapham P.R., Ostroff G.R. (2016). Targeted Delivery of Glucan Particle Encapsulated Gallium Nanoparticles Inhibits HIV Growth in Human Macrophages. J. Drug Deliv..

[82] Wang D., Gao C., Si T., Li Z., Guo B., He Q. (2020). Near-infrared light propelled motion of needlelike liquid metal nanoswimmers. Colloids Surf. A Physicochem. Eng. Asp..

[83] Yuan B., Tan S.C., Zhou Y.X., Liu J. (2015). Self-powered macroscopic Brownian motion of spontaneously running liquid metal motors. Sci. Bull..

[84] Zhang J., Guo R., Liu J. (2016). Self-propelled liquid metal motors steered by a magnetic or electrical field for drug delivery. J. Mater. Chem. B.

[85] Tan S.C., Yuan B., Liu J. (2015). Electrical method to control the running direction and speed of self-powered tiny liquid metal motors. Proc. R. Soc. Math. Phys. Eng. Sci..

[86] Tan S.C., Gui H., Yuan B., Liu J. (2015). Magnetic trap effect to restrict motion of self-powered tiny liquid metal motors. Appl. Phys. Lett..

[87] Mohammed M.G., Xenakis A., Dickey M.D. (2014). Production of liquid metal spheres by molding. Metals.

[88] Thelen J., Dickey M.D., Ward T. (2012). A study of the production and reversible stability of EGaIn liquid metal microspheres using flow focusing. Lab Chip.

[89] Tang S.Y., Joshipura I.D., Lin Y., Kalantar-Zadeh K., Mitchell A., Khoshmanesh K., Dickey M.D. (2016). Liquid-Metal Microdroplets Formed Dynamically with Electrical Control of Size and Rate. Adv. Mater..

[90] Friedman H., Porat Z.E., Halevy I., Reich S. (2011). Formation of metal microspheres by ultrasonic cavitation. J. Mater. Res..

[91] Kumar V.B., Koltypin Y., Gedanken A., Porat Z. (2014). Ultrasonic cavitation of molten gallium in water: Entrapment of organic molecules in gallium microspheres. J. Mater. Chem. A.

[92] Deng B., Cheng G.J. (2019). Pulsed Laser Modulated Shock Transition from Liquid Metal Nanoparticles to Mechanically and Thermally Robust Solid–Liquid Patterns. Adv. Mater..

[93] MacDonald K.F., Fedotov V.A., Pochon S., Ross K.J., Stevens G.C., Zheludev N.I., Brocklesby W.S., Emel’Yanov V.I. (2002). Optical control of gallium nanoparticle growth. Appl. Phys. Lett..

[94] Yarema M., Wörle M., Rossell M.D., Erni R., Caputo R., Protesescu L., Kravchyk K.V., Dirin D.N., Lienau K., von Rohr F. (2014). Monodisperse colloidal gallium nanoparticles: Synthesis, low temperature crystallization, surface plasmon resonance and Li-ion storage. J. Am. Chem. Soc..

[95] Gao W., Pei A., Wang J. (2012). Water-Driven Micromotors. ACS Nano.

[96] Zhang J., Yao Y., Sheng L., Liu J. (2015). Self-fueled biomimetic liquid metal mollusk. Adv. Mater..

[97] Zavabeti A., Daeneke T., Chrimes A.F., O’Mullane A.P., Ou J.Z., Mitchell A., Khoshmanesh K., Kalantar-Zadeh K. (2016). Ionic imbalance induced self-propulsion of liquid metals. Nat. Commun..

[98] Tang J., Wang J., Liu J., Zhou Y. (2016). Jumping liquid metal droplet in electrolyte triggered by solid metal particles. Appl. Phys. Lett..

[99] Liu H., Li M., Li Y., Yang H., Li A., Lu T.J., Li F., Xu F. (2018). Magnetic steering of liquid metal mobiles. Soft Matter.

[100] Shu J., Tang S.Y., Feng Z., Li W., Li X., Zhang S. (2018). Unconventional locomotion of liquid metal droplets driven by magnetic fields. Soft Matter..

[101] Shu J., Tang S.-Y., Zhao S., Feng Z., Chen H., Li X., Li W., Zhang S. (2019). Rotation of Liquid Metal Droplets Solely Driven by the Action of Magnetic Fields. Appl. Sci..

[102] Tang S.Y., Sivan V., Khoshmanesh K., O’Mullane A.P., Tang X., Gol B., Eshtiaghi N., Lieder F., Petersen P., Mitchell A. (2013). Electrochemically induced actuation of liquid metal marbles. Nanoscale.

[103] Paxton W.F., Baker P.T., Kline T.R., Wang Y., Mallouk T.E., Sen A. (2006). Catalytically induced electrokinetics for motors and micropumps. J. Am. Chem. Soc..

[104] Wang Y., Hernandez R.M., Bartlett D.J., Bingham J.M., Kline T.R., Sen A., Mallouk T.E. (2006). Bipolar electrochemical mechanism for the propulsion of catalytic nanomotors in hydrogen peroxide solutions. Langmuir.

[105] Wang M.F., Jin M.J., Jin X.J., Zuo S.G. (2017). Modeling of movement of liquid metal droplets driven by an electric field. Phys. Chem. Chem. Phys..

[106] Erikson K.R., Fry F.J., Jones J.P. (1974). Ultrasound in Medicine—A Review. IEEE Trans. Sonics Ultrason..

[107] Ziskin M.C., Petitti D.B. (1988). Epidemiology of human exposure to ultrasound: A critical review. Ultrasound Med. Biol..

[108] Litvak E., Foster K.R., Repacholi M.H. (2002). Health and safety implications of exposure to electromagnetic fields in the frequency range 300 Hz to 10 MHz. Bioelectromagnetics.

[109] Barnett S.B., Ter Haar G.R., Ziskin M.C., Rott H.-D., Duck F.A., Maeda K. (2000). International recommendations and guidelines for the safe use of diagnostic ultrasound in medicine. Ultrasound Med. Biol..

[110] Wang W., Chiang T.-Y., Velegol D., Mallouk T.E. (2013). Understanding the Efficiency of Autonomous Nano- and Microscale Motors. J. Am. Chem. Soc..

[111] Wang W., Castro L.A., Hoyos M., Mallouk T.E. (2012). Autonomous motion of metallic microrods propelled by ultrasound. ACS Nano.

[112] Ahmed S., Wang W., Mair L.O., Fraleigh R.D., Li S., Castro L.A., Hoyos M., Huang T.J., Mallouk T.E. (2013). Steering acoustically propelled nanowire motors toward cells in a biologically compatible environment using magnetic fields. Langmuir.

[113] Garcia-Gradilla V., Sattayasamitsathit S., Soto F., Kuralay F., Yardimci C., Wiitala D., Galarnyk M., Wang J. (2014). Ultrasound-propelled nanoporous gold wire for efficient drug loading and release. Small.

[114] De Castro A.I., Chrimes A.F., Zavabeti A., Berean K.J., Carey B.J., Zhuang J., Du Y., Dou S.X., Suzuki K., Shanks R.A. (2017). A Gallium-Based Magnetocaloric Liquid Metal Ferrofluid. Nano Lett..

[115] Tang J., Zhao X., Li J., Zhou Y., Liu J. (2017). Liquid Metal Phagocytosis: Intermetallic Wetting Induced Particle Internalization. Adv. Sci..

[116] Hu L., Wang H., Wang X., Liu X., Guo J., Liu J. (2019). Magnetic Liquid Metals Manipulated in the Three-Dimensional Free Space. ACS Appl. Mater. Interfaces.

[117] Tang X.K., Tang S.Y., Sivan V., Zhang W., Mitchell A., Kalantar-zadeh K., Khoshmanesh K. (2013). Photochemically induced motion of liquid metal marbles. Appl. Phys. Lett..

[118] Wang W., Wu Z., He Q. (2020). Swimming nanorobots for opening a cell membrane mechanically. View.

[119] Hou Y., Lu C., Dou M., Zhang C., Chang H., Liu J., Rao W. (2020). Soft liquid metal nanoparticles achieve reduced crystal nucleation and ultrarapid rewarming for human bone marrow stromal cell and blood vessel cryopreservation. Acta Biomater..

[120] Wang W., Duan W., Ahmed S., Sen A., Mallouk T.E. (2015). From one to many: Dynamic assembly and collective behavior of self-propelled colloidal motors. Acc. Chem. Res..

[121] Xia N., Li N., Rao W., Yu J., Wu Q., Tan L., Li H., Gou L., Liang P., Li L. (2019). Multifunctional and flexible ZrO2-coated EGaIn nanoparticles for photothermal therapy. Nanoscale.

